# Integrated NMR and cryo-EM atomic-resolution structure determination of a half-megadalton enzyme complex

**DOI:** 10.1038/s41467-019-10490-9

**Published:** 2019-06-19

**Authors:** Diego F. Gauto, Leandro F. Estrozi, Charles D. Schwieters, Gregory Effantin, Pavel Macek, Remy Sounier, Astrid C. Sivertsen, Elena Schmidt, Rime Kerfah, Guillaume Mas, Jacques-Philippe Colletier, Peter Güntert, Adrien Favier, Guy Schoehn, Paul Schanda, Jerome Boisbouvier

**Affiliations:** 1 Institut de Biologie Structurale (IBS), CEA, CNRS, Université Grenoble Alpes, 71, Avenue des Martyrs, F-38044 Grenoble, France; 2 Laboratory of Imaging Sciences, Center for Information Technology, National Institutes of Health, 12 South Drive, MSC 5624, Bethesda, MD 20892 USA; 3 Institute of Biophysical Chemistry, Goethe University Frankfurt am Main, 60438 Frankfurt am Main, Germany; 4 Laboratory of Physical Chemistry, ETH Zürich, 8093 Zürich, Switzerland; 5 Graduate School of Science, Tokyo Metropolitan University, Tokyo 192-0397, Japan; 6 Present Address: NMR-Bio, 5 Place Robert Schuman, F-38025 Grenoble, France; 7 Present Address: Institut de Génomique Fonctionnelle, CNRS UMR-5203, INSERM U1191, University of Montpellier, F-34000 Montpellier, France; 8 Present Address: Biozentrum University of Basel, Klingelbergstrasse 70, 4056 Basel, Switzerland

**Keywords:** Cryoelectron microscopy, NMR spectroscopy, Solid-state NMR

## Abstract

Atomic-resolution structure determination is crucial for understanding protein function. Cryo-EM and NMR spectroscopy both provide structural information, but currently cryo-EM does not routinely give access to atomic-level structural data, and, generally, NMR structure determination is restricted to small (<30 kDa) proteins. We introduce an integrated structure determination approach that simultaneously uses NMR and EM data to overcome the limits of each of these methods. The approach enables structure determination of the 468 kDa large dodecameric aminopeptidase TET2 to a precision and accuracy below 1 Å by combining secondary-structure information obtained from near-complete magic-angle-spinning NMR assignments of the 39 kDa-large subunits, distance restraints from backbone amides and ILV methyl groups, and a 4.1 Å resolution EM map. The resulting structure exceeds current standards of NMR and EM structure determination in terms of molecular weight and precision. Importantly, the approach is successful even in cases where only medium-resolution cryo-EM data are available.

## Abstract (web summary)

NMR structure determination is challenging for proteins with a molecular weight above 30 kDa and atomic-resolution structure determination from cryo-EM data is currently not the rule. Here the authors describe an integrated structure determination approach that simultaneously uses NMR and EM data and allows them to determine the structure of the 468 kDa dodecameric aminopeptidase TET2 complex.

## Introduction

For decades, the weight of atomic-resolution structure elucidation, a requirement for understanding biomolecular mechanisms in detail, has been almost exclusively borne by X-ray crystallography, as highlighted by the fact that about 90% of all entries in the Protein Data Bank (PDB) are crystal structures. Notwithstanding this success of crystallography, many supra-molecular edifices, self-assembling systems, membrane proteins, and proteins with extended dynamic domains are difficult to crystallize, or the crystals do not diffract to high resolution. Single-particle cryo electron microscopy (cryo-EM) and nuclear magnetic resonance spectroscopy (NMR) are not bound to obtaining well-ordered crystals, and are applicable even in the presence of significant motions. Enabled by decisive instrumental and methodological progress, cryo-EM has recently made a leap in resolution^1,2^, which opened avenues for determining protein structures at atomic detail. Nonetheless, de novo atomic-resolution structure determination from cryo-EM data is currently not the rule. A survey of the EM maps deposited in the EM data base (EMDB) over the last 2 years reveals an average resolution in the range of 6.5–8 Å. In 2016, a particularly productive year for single-particle cryo-EM (961 entries in the EMDB), only 20% of the deposited maps had a resolution of 3.5 Å or better, thus being well suited for atomic-level de novo structure determination. Extending EM structure determination to a wider range of biological objects may require further resolution increase and/or combination of EM with other data.

NMR spectroscopy probes the structure of proteins on a length scale that is very different from that seen by EM. Rather than probing the electronic potential of the molecule, providing a more or less well-defined molecular envelope, NMR detects the immediate vicinity of hundreds to thousands of individual atomic nuclei across the protein. Their resonance frequency is exquisitely sensitive to the local electronic environment and, thus, to the local structure. Furthermore, dedicated NMR experiments identify which atoms are in the vicinity (<5–10 Å) of a given atomic nucleus, thus allowing to assemble structural elements into a three-dimensional structure. The precision and accuracy of NMR-derived structures strongly depend on the availability of a large number of such distance restraints across the entire molecule. When NMR structure determination fails, it is generally the paucity of meaningful distance restraints—often as a consequence of resonance overlap or low detection sensitivity—which hampers the arrangement of local structural elements to a well-defined tertiary fold. In solution-state NMR, structure determination is particularly challenging for proteins larger than 50 kDa, as the slow molecular tumbling leads to rapid nuclear relaxation and thereby low detection sensitivity, which results in difficulties obtaining information about local conformation and distances. Consequently, besides three cases^3–5^ the PDB is, to our knowledge, devoid of de novo NMR structures beyond 50 kDa (subunit size). The introduction of deuteration and specific methyl labeling with ^13^CH_3_ groups has greatly expanded the molecular weight range in which solution-NMR signals can be detected to ca. 1 MDa^6–8^. Yet because this approach is limited to methyl groups, no information about the backbone conformation can be obtained, which excludes any de novo structure determination.

Magic-angle spinning (MAS) solid-state NMR, overcomes the inherent size limitations that its solution-state counterpart faces by replacing the slow stochastic molecular tumbling in solution—which is at the origin of the rapid NMR signal loss—by immobilization of the protein, and spinning of the sample at a constant “magic” angle^9^. Recent advances in MAS NMR sample preparation, including optimized isotope-labeling schemes, hardware allowing fast sample spinning (>50 kHz), and optimized pulse sequences have enabled the structure determination of small to medium-sized proteins forming crystals, oligomeric assemblies, or embedded in membranes, generally with monomer sizes below 20 kDa^10^, with an exceptional case at 27 kDa^11^. Sensitivity limitations and the increased spectral complexity and signal overlap in large proteins have so far been a bottleneck for the extraction of sufficient useful restraints for structure determination on larger proteins.

While EM and NMR spectroscopy probe protein structure on different length scales, these complementary views may be combined. Recent work has explored the joint use of NMR and EM data to determine protein structures^12–14^. The proteins studied are small (<100 residues) and comprise only 2 or 3 secondary structure elements, which made it possible to either first determine a global fold from NMR and refine this model with the aid of the EM map^12^, or to build a first model into the Coulombic potential map, and use NMR data to refine these manually constructed EM models^13,14^. For larger proteins, the situation is significantly more complex: the former approach, i.e., determining a rough fold from NMR and then refining with EM data, is challenging because lack of NMR restraints—often the case for large proteins—precludes obtaining a converged structural model which then could be used for EM-based refinement. The latter method suffers from the fact that de novo building of the protein sequence into an EM map, i.e., unambiguously identifying the residues and being able to follow the chain throughout the map, becomes increasingly complex with protein size, and very strongly depends on the resolution of the EM data.

Here, we present an approach that overcomes these challenges and combines NMR and EM information in a joint and lightly-supervised manner. Briefly, the key ingredients for the combined analysis from these two techniques are (i) the identification of structural features (such as α-helices) in 3D space from cryo-EM maps, (ii) the NMR-based identification of these structural elements along the sequence, in particular the residue-wise assignment of secondary structures, (iii) the unambiguous assignment of these sequence stretches to 3D structural features detected by EM, guided by NMR-derived distance restraints, and (iv) the joint refinement of the protein structure against NMR data and an electron-density map.

We apply the integrated NMR/EM approach to the dodecameric 12 × 39 kDa (468 kDa) large aminopeptidase enzyme TET2 from *Pyrococcus horikoshii* which forms a large hollow lumen encapsulating 12 peptidase active sites^15,16^. We use cryo-EM maps with resolutions of 4.1, 6, and 8 Å (the latter two were obtained by Fourier-space truncation), as well as secondary structure and distance information from MAS NMR and solution-NMR. Despite its large subunit size of 353 residues, larger than any protein assigned to date by MAS NMR, we achieved a near-complete NMR resonance assignment in a reliable manner, which allows the residue-wise determination of the backbone conformation, as well as hundreds of experimental distance restraints involving the protein backbone and side chain methyl groups, but by themselves these data are insufficient for structure determination. An automated approach that jointly exploits the EM map, NMR secondary structure, and distance restraints is presented, that allowed us to obtain the structure of TET2 at a backbone root-mean-square-deviation (RMSD), of 0.7 Å to the crystal structure^15,16^.

Importantly, we show that the NMR/EM approach provides near-atomic-resolution structures even if only moderate-resolution EM data are available, an often-encountered situation that prohibits structure determination from EM data alone (probed here by Fourier-space truncation to 8 Å resolution). Our structure provides insight into regions that had not been visible in previous crystal structures of TET2, in particular a conserved loop region in the catalytic chamber of this enzyme machinery.

## Results

### NMR and EM: structure information on different length scales

**Fig. 1:**
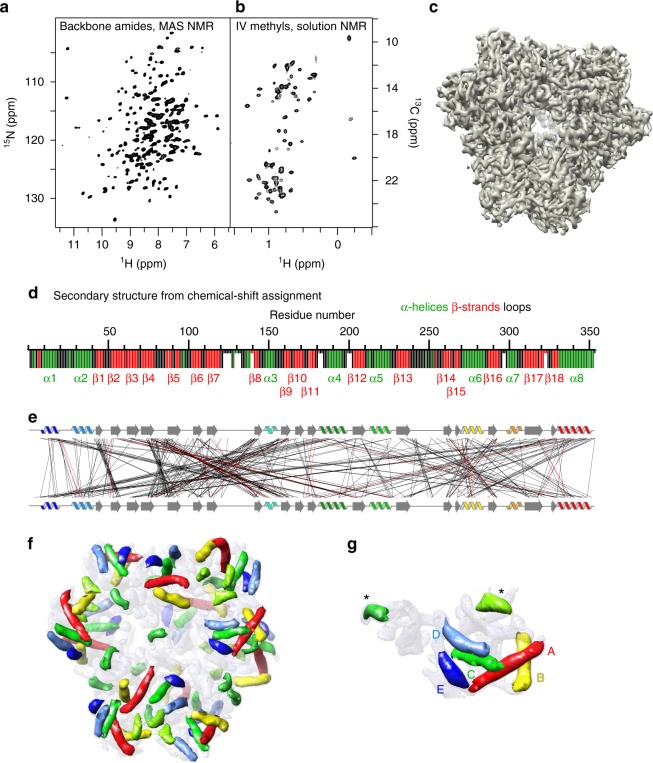
Experimental NMR and EM data of the 468 kDa TET2 assembly. **a** MAS NMR and **b** solution-NMR spectra of TET2, showing high resolution despite the large subunit size. **c** Experimental 4.1 Å resolution cryo-EM electron-density map. **d** Secondary structure of TET2, derived from MAS NMR resonance assignments and the TALOS-N software^20^, shown as a function of the residue number. Residues shown with shorter bars were not assigned, and the secondary structure assignment results from a database approach in TALOS-N. **e** Experimentally detected intra-subunit long-range distance restraints from solution-NMR and MAS NMR, displayed through lines connecting residues in close spatial proximity. Note that part of these distance restraints were spectrally ambiguous, i.e., could be assigned to several atom-atom pairs, and was rendered unambiguous throughout the structure calculation approach (displayed in red). See Table 1 for restraints statistics. All NMR experiments performed in this study and acquisition parameters are listed in Supplementary Tables 2–5. **f** α-Helices detected by the helixhunter2 software^25,50^ in the EM map truncated to 8 Å resolution. Symmetry-related α-helices are shown in equal colors. Additional β-sheet parts, automatically detected by gorgon^51^ are shown in Supplementary Fig. 8A. **g** Zoom on one subunit, identified by a clustering analysis (Supplementary Fig. 8). The five longest α-helices, used for the initial structure calculation steps are labeled with A to E in order of decreasing length (see Table 1)

Any investigation of structure, interactions, or dynamics by NMR-based methods is bound to the availability of well-resolved spectra and the ability to assign the observed peaks to individual atoms along the protein sequence. Figure 1a, b shows MAS NMR spectra of the protein backbone amides and solution-NMR of Ile and Val methyl groups in TET2, respectively. Despite the large number of residues, 353 residues per subunit, and the expected large number of signals, we obtained well-resolved spectra at high sensitivity. The 12-fold symmetry results in a single set of cross-peaks for all subunits in the 468 kDa-large particle. In solution, methyl spectra can be observed that are very similar to the corresponding MAS NMR spectra obtained. This shows that the sample states are structurally comparable, which allows combining the data from solution-NMR and MAS NMR in a straightforward manner (see Supplementary Fig. 1). To obtain atom-specific assignments of these spectra we collected a series of three- and four-dimensional ^13^C-detected and ^1^H-detected MAS NMR assignment spectra on uniformly labeled TET2 MAS NMR samples, which correlate the frequencies of neighboring atoms along the backbone and into the side chains, thereby allowing to connect them along the protein backbone and out into the side chains.

Additional MAS NMR spectra recorded on three different samples with amino-acid-type specific ^13^C-labeling (either only LKP (Leu, Lys, Pro), GYFR or ILV residues) provided simplified spectra, which served as convenient starting points for manual assignment (see Methods and Supplementary Tables 2–4 for details). Together, these MAS NMR spectra were sufficient to obtain by manual analysis a near-complete assignments of TET2, ca. 85% of the backbone and 70% of the side chain heavy atoms (Supplementary Fig. 2). Residues for which no assignments were obtained are restricted to N- and C-termini and loops, a finding that can be attributed to the lower efficiency of the MAS NMR experiments for such dynamic parts. The assignment of 94 Ile, Leu, Val methyl groups was achieved through a combination of a mutagenesis-based strategy in solution, reported previously^17,18^, and the ^13^C frequencies obtained from MAS NMR assignment experiments. Even though TET2 is larger than any protein assigned to date to a similar extent by MAS NMR, the approach is sufficiently robust to obtain the near-complete assignment even in a fully automatic manner with the program FLYA^19^ (Supplementary Fig. 3), showing that reliable assignments can be obtained without time-consuming manual spectra analysis. The assigned chemical shifts from MAS NMR allow the determination of $\phi$ and $\psi$ backbone dihedral angles, and thus the secondary structure from a database approach, TALOS-N^20^. NMR-detected secondary structure elements are displayed in Supplementary Fig. 2E.

In order to gain tertiary structure information, we measured short inter-atomic contacts through (i) solution-NMR 3D nuclear Overhauser effect spectroscopy (NOESY) spectra and (ii) a 3D MAS NMR radio-frequency driven recoupling (RFDR) experiment (Supplementary Fig. 4). The solution-NMR data identify spatial proximity between Ile^*δ*1^ and Val^proS^ methyl groups distant by up to 8 Å^21^; a second NOESY spectrum, recorded on a sample in which different subunits were labeled in different methyl groups, furthermore allowed filtering out inter-subunit methyl–methyl contacts (Supplementary Fig. 4E–G). The MAS NMR experiment provides distances between backbone amide sites and between amides and methyls of Ile, Val, and Leu side chains, as well as contacts between such methyls in a single time-shared ^13^C/^15^N 3D MAS NMR experiment (Supplementary Fig. 4). ^13^C–^13^C distances were additionally measured in the selectively LKP-labeled sample by MAS NMR. Figure 1e and Table 1 show all distance restraints identified by NMR, including 471 spectrally unambiguous ones, i.e., of which the cross-peak frequencies pointed unambiguously to one atom pair and 45 spectrally ambiguous ones, i.e., which could not be assigned to a single atom pair (but which could be unambiguously assigned along the structure calculation procedure, see below). The majority of these restraints (all 544 backbone dihedral restraints and ca. 80% of the distance restraints) was obtained from MAS NMR.

**Table 1:** NMR restraints and structural quality assessment

Structural restraints (all intra-subunit)	
Total distance restraints (calculation set)	516
Short-range (|*i* − *j*| < 4)	203
Long-range (|*i* − *j*| ≥ 4)	313
Unambiguous H–H	441
Unambiguous C–C	30
Ambiguous H–H	45
Dihedral angle restraints ($\phi$, $\psi$)	544
Statistics NMR + EM, after step 3 (PDB 6F3K)	
Ramachandran most favored regions	93 ± 1%
Allowed regions	4 ± 1%
Outliers	3 ± 1%
Ensemble backbone precision (Å)	0.29
CC_mask_	0.67
Statistics after additional Phenix refinement (PDB 6R8N)	
Ramachandran most favored regions	90%
Allowed regions	10%
Outliers	0%
CC_mask_	0.73

Attempts to determine the TET2 subunit structure from these NMR data using a standard structure-determination protocol based on restrained MD, CYANA^22^, failed in achieving convergence to the correct structure (backbone RMSD to the mean of the ten lowest-energy structures and to the crystal structure above 10 Å, see Supplementary Fig. 5). In these NMR-only structure calculations, many local structural elements, e.g., the local packing of two α-helices or β-strands, are formed, but the positions of these elements relative to each other in the tertiary fold remained poorly defined, thus calling for additional data.

Figure 1c shows the cryo-EM map of TET2 at 4.1 Å resolution, calculated from 27,130 single particles with the software RELION (see Methods section for details and Supplementary Fig. 6). We have attempted to build the structure of TET2 from this cryo-EM map using phenix.model_to_map^23^, in the crystallographic software suite Phenix^24^. The program was able to trace the map, with better success when the symmetry of the particle was calculated from the cryo-EM map rather than inferred from the biological unit derived from the X-ray structure. However, the map quality was too low for Phenix to assign the sequence and thus build a model (Supplementary Fig. 7). It may have been possible to solve the structure at 4.1 Å by careful manual building, or by using other computational tools. Nonetheless, the fact that Phenix, an advanced and widely used program for model building, fails in our case illustrates that even with 4.1 Å resolution, de novo model building is far from trivial. This finding also highlights that the likelihood of succeeding with model building rapidly decays with decreasing resolution. The collection of a larger data set may have allowed obtaining EM data at near-atomic resolution. Nonetheless, at 4.1 Å resolution, significantly better than the average of EM maps deposited over the last years, our EM data set is insufficient for de novo structure determination, highlighting the challenges that many EM data sets encounter.

Despite the inability to trace the backbone chain in the density, a number of features, in particular α-helices, are readily recognized in the cryo-EM map, even at lower resolution and in a fully automatic lightly-supervised manner^25^. Figure 1f, g shows the 12 × 7 α-helices that could be automatically detected from the cryo-EM map of TET2 truncated to 8 Å resolution and Supplementary Fig. 8A shows additionally detected β-sheet regions. Although the data set truncated to 8 Å resolution is arguably better than a “real-life” data set at 8 Å, it is clear that such structural features are readily recognized in many medium-resolution data sets. NMR and EM thus provided structural information about TET2 on very different length scales, but neither of the two techniques by themselves allowed to determine the structure at the atomic scale, owing to the lack of long-range distance information in NMR, and the inability to build a robust model from the EM map.

### Automated integrated EM/NMR structure calculation

We developed an approach for the combined use of EM and NMR data, keeping in mind that it should be applicable to cases where only medium-resolution cryo-EM maps (between 6 and 10 Å) would be available. Its central idea is the fact that certain structural elements can be identified even in medium-resolution EM maps and by unsupervised automatic algorithms. The arrangement of these structural elements in 3D space, i.e., the knowledge of the positions of even a small subset of residues relative to each other, potentially brings very useful long-range structural information, and it is exactly such long-range information that is very valuable in NMR-based structure calculations.

Placing sequence stretches into the density can be viewed as a combinatorial assignment problem, in which sequence elements with known properties (e.g., α-helices, β-strands, or large side chains that can be recognized in EM maps) are assigned to electron-density features with the matching shape. The most straightforward case is the one of α-helices, given the ease of detecting the α-helical electron-density features automatically, and assigning α-helical structures to sequence stretches from NMR data (Fig. 1d, f, g, respectively).

**Fig. 2:**
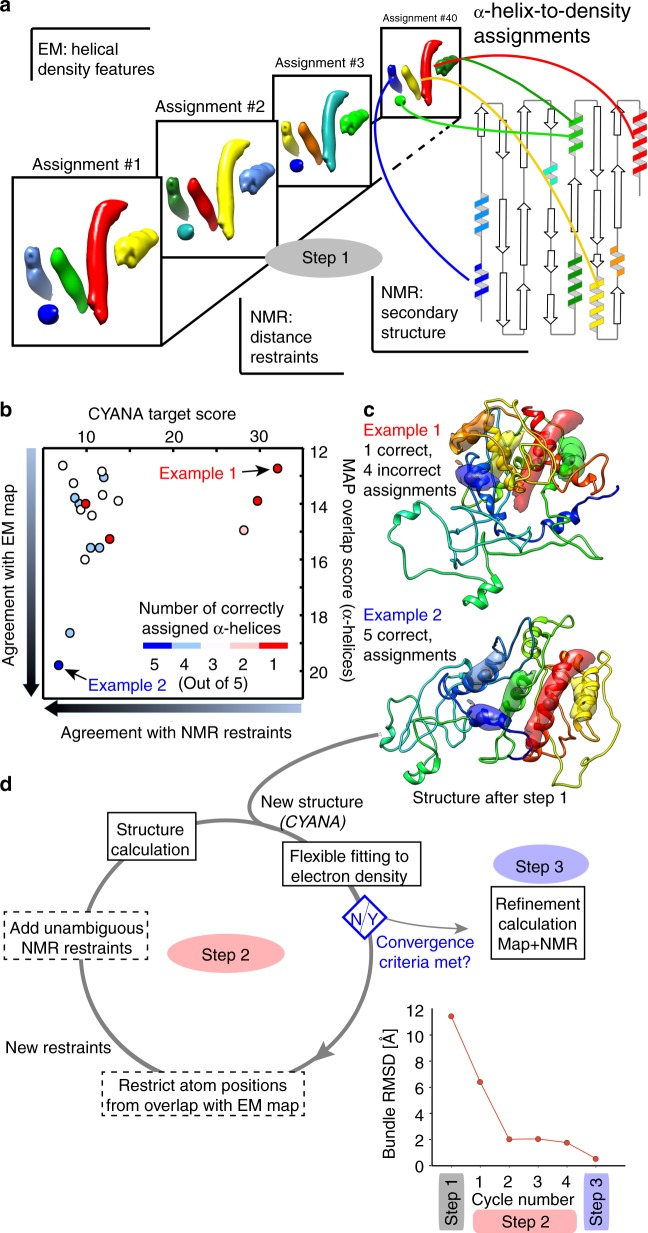
Integrated NMR/EM structure determination approach. **a** In step 1, 40 different assignment possibilities of the five longest helical stretches in the sequence (TALOS) to cylindrical (helical) densities (“α-helix-to-density assignments”) are used in regular NMR-type structure calculations (CYANA). **b** Ranking of the 20 solutions from these structure calculations by the CYANA target score and the overlap of the α-helices with the cylindrical density features. Each point represents the average of the 10 lowest-energy structures. **c** Two example cases are shown with incorrect (top) and correct (bottom) assignments, clearly showing that incorrect α-helix-to-density assignment is not compatible with good map overlap. For simplicity, only the lowest-energy structure is shown. The structure with the correct helix-to-density assignment (labeled as Example 2) has a backbone root-mean-square-deviation, RMSD, to the crystal structure of 7.4 Å, and a backbone bundle-RMSD computed from the 20 lowest-energy models of 11.4 Å. **d** In step 2, the structure of the TET2 monomeric subunit with the correct α-helix-to-density assignment is iteratively refined by flexible fitting into the EM map (truncated at 8 Å resolution), and CYANA calculations with an increasing number of unambiguous NMR restraints and restraints from the fit in the EM map. After convergence, defined by at least three cycles with an RMSD-difference below 10% (shown in (**e**)), the structure is refined against the EM map of the entire dodecameric ensemble and all NMR restraints, using XPLOR-NIH, using maps with increasing resolution (8, 6, 4.1 Å; step 3). **e** Root-mean-square-deviation (RMSD) of the structures at different steps of the protocol, relative to the mean structure

Briefly, our approach, outlined in Fig. 2, consists of three steps. In the first step, after detection of the α-helices in the EM map and in the sequence, the various possibilities of placing α-helical sequence stretches into α-helix density features are systematically and combinatorially tested, performing with each of these helix-to-density assignments a restrained-MD type structure calculation with CYANA. This type of calculation follows standard procedures adopted in any NMR-based structure calculation, but the key difference is that in addition to NMR-derived distance restraints the α-helices are placed at precisely defined positions relative to each other. Subsequently, the correct helix-to-density assignment is identified through objective criteria that compare the resulting structural models to experimental NMR and EM observables. With the correct helix-to-density assignment at hand, steps 2 and 3 of our approach refine this model by iterative addition of restraints from the cryo-EM map as well as from NMR distance restraints that could be unambiguously assigned based on the initial structural models (see below).

An important consideration for the combinatorial procedure of step 1 is that the number of possible helix-to-density assignment combinations increases exponentially with the number of α-helices. Considering that the eight α-helices found in the sequence by NMR can be placed into the automatically detected helix features in the map (seven per subunit), including two orientations in each helix, there are 8 × 2^7^ ≈ 5 · 10^6^ possibilities (see Supplementary Table 1 and discussion therein). Even more combinations are possible when considering that it is not straightforward to unambiguously identify which helical densities belong to the same subunit. Performing structural calculations with each of these assignments would be computationally very expensive.

A computationally manageable number of assignments can be achieved by taking into account some simple experimental observables. First, we used cluster analysis to identify α-helical densities which are closest in space as belonging to the same asymmetric unit, and ascribed these five helical densities to the same subunit (see Fig. 1g and Supplementary Fig. 8), and used only five helices, discarding at this step the two shortest ones that had been also identified in the EM map. Furthermore, we explicitly disregarded the polarity when assigning helical stretches to densities, by enforcing that only the central residue of a given α-helical sequence stretch is fixed to the center of an α-helical density, rather than the entire helix. Lastly, we discarded all those assignments for which the lengths of the α-helices along the sequence did not match the length of the helical density (see Supplementary Table 1). With these assumptions and simplifications, only 40 possible assignments remain and we performed 40 CYANA structure calculations with these helix-to-density assignments (five residues in the centers of helices restrained at fixed positions relative to each other; see Supplementary Table 1), along with the backbone dihedral-angle and the spectrally unambiguous NMR distance restraints.

Identification of the correct helix-to-density assignment was based on two criteria, (i) the CYANA target function, which reflects the agreement of the structure with the distance and dihedral-angle restraints, and (ii) the overlap of the five α-helical stretches with the cryo-EM map. Incorrect helix-to-density assignments generally result in bent or twisted α-helices, and thus low overlap with the EM map and violation of NMR distances. Figure 2b displays these two scores for the 20 CYANA runs with the lowest CYANA score. A funnel-shaped distribution is observed for the various structures. Figure 2c displays two of these structures, in which either one or five α-helices were assigned correctly (out of five helices that were considered). The structure with the correct helix-to-density assignment scores best among all structures (denoted as “Example 2” in Fig. 2b, c) . While it has the topology approximately correct, its precision and accuracy are low, calling for further refinement steps.

**Fig. 3:**
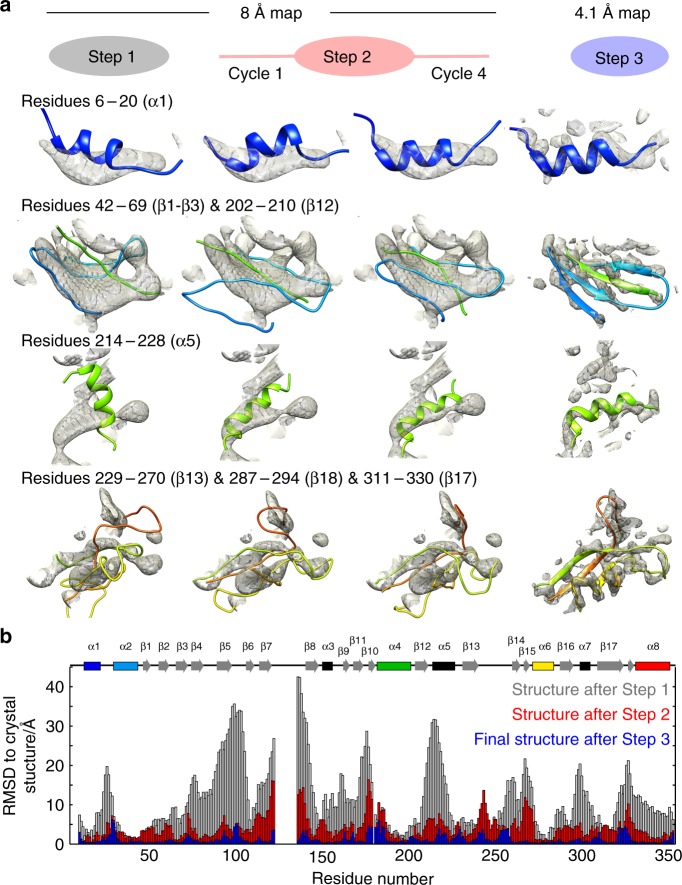
Structure refinement from NMR and EM data. **a** Zoom onto selected parts of the TET2 structure along the different steps of the structure determination process. The 3D EM map used at 8 Å resolution (steps 1 and 2) and 4.1 Å resolution (step 3) is shown as a gray surface. The structures shown correspond to the lowest-energy models generated by CYANA (steps 1 and 2) or XPLOR-NIH (step 3). A comprehensive view of the evolution of all structure elements is provided in Supplementary Fig. 11. **b** Residue-wise heavy-atom backbone RMSD relative to the crystal structure throughout different steps of the structure determination protocol

**Fig. 4:**
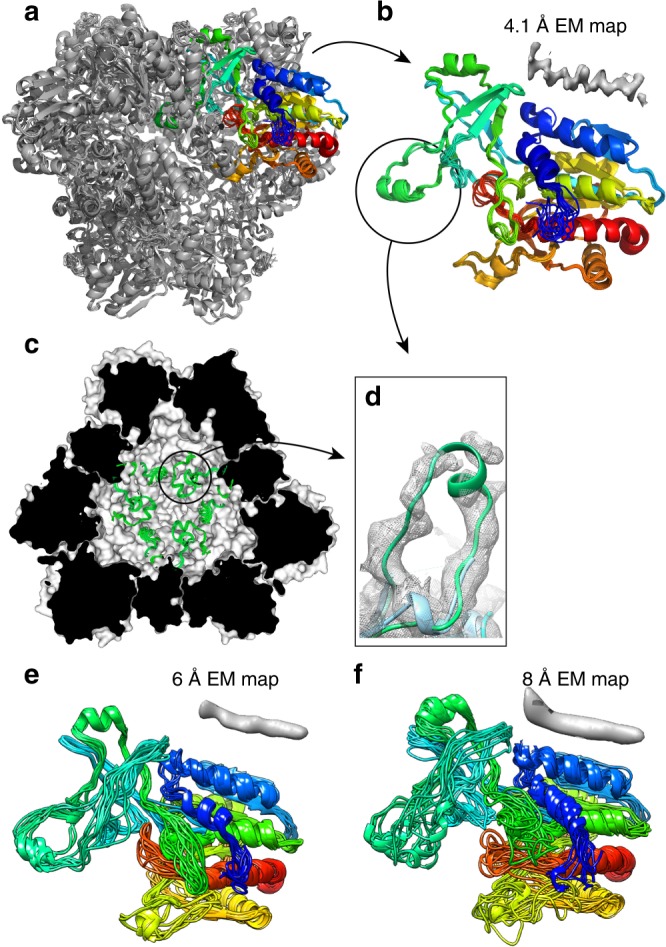
Structure of the TET2 dodecamer from EM/NMR. **a** Overall view of the dodecamer, highlighting in color one out of the 12 modeled subunits, obtained from joint refinement of 12 subunits against NMR and EM data. **b** Bundle of 10 structures of one monomer, with an accuracy of 0.7 Å (backbone RMSD relative to crystal structure). The loop region, residues 120–138, which has not been modeled in the crystal structure (PDB 1y0r) is encircled in (**b**), and shown in the view of the interior of the cavity (**c**) and as a zoom (**d**). The EM map around the loop region, seen by cryo-EM (**d**) is of significantly higher intensity than in the 100 K crystal structure (1y0r). The backbone structure in both panels is from the present NMR/EM study. **e**, **f** Results of NMR/EM structure calculation using only EM data truncated to 6 or 8 Å resolution, resulting in backbone RMSD to the crystal structure of 1.8 and 2.6 Å, respectively. The inserts above the structures in (**b**, **e**, **f**) represent typical α-helical densities at the respective resolution levels

The refinement stage of the protocol, an automated iterative procedure denoted here as step 2 (Fig. 2d), consists of iterative cycles of (i) a flexible fitting of the initial structure into the 8 Å EM density with iMODFIT^26^, and (ii) CYANA structure calculations. In each iteration, EM and NMR restraints were added: on the one hand, sequence stretches consistently located inside the 8 Å EM map were restrained to their positions, in the same manner as the helix-centers were initially fixed relative to each other (see Supplementary Fig. 9); on the other hand, the initial structural models allowed the unambiguous assignment of more NMR distance restraints, by excluding assignment possibilities corresponding to physically unrealistic long-range distances (see Methods). This iterative procedure was repeated until convergence of the bundle-RMSD (Fig. 2e). In step 3, a real-space structure refinement was applied in the XPLOR-NIH software^27,28^, using the full Coulombic potential map, first at 8 Å, then at 6 Å and lastly at 4.1 Å resolution, and all NMR restraints, calculating the full dodecamer. Figure 3 shows the evolution of the structure from the initial model to the final structure, which was obtained with high precision (bundle backbone RMSD over the 20 lowest-energy structures of 0.29 Å for one subunit). As with any structure determination protocol, validation of the structure is important. In the general case, validation may comprise consistency checks against independent NMR data that have been left out from the calculation, or correlation with the EM map. In the case of TET2, a crystal structure was available, and the refined dodecameric structure (Fig. 4) is in excellent agreement with the crystal structure and the EM map (Supplementary Fig. 10B).

The dodecameric assembly forms a tetrahedral structure with ca. 12 Å wide openings, one on each of the four tetrahedron faces, providing access to the ca. 50 Å wide catalytic lumen. Our structure features a long loop region in the catalytic chamber, comprising residues 119–138, shown in green in Fig. 4c, d. This loop has not been modeled in the crystal structure. Moreover, while this loop region appears relatively well defined at cryo-EM conditions, although at lower local resolution, only methyl resonances of Val-120 and Ile-124 were detected by NMR but we were unable to assign the backbone resonances of this loop at room temperature by MAS NMR (Supplementary Fig. 2E; the short helix we modeled was inferred from the TALOS-N algorithm.) These findings may suggest that the loop is dynamically disordered at room temperature, leading to low signal intensity in MAS NMR experiments, and to non interpretable density in crystal structures determined at cryogenic temperatures and weaker intensity in the Coulombic potential map from EM for this region. Possibly, interactions between residues in neighboring loops (residues 127–131) found to be in proximity in the EM map might stabilize the loop conformation, leading to a reduced but still visible density at cryogenic temperatures.

An important consideration is how our approach performs with lower-resolution EM data. At 4.1 Å resolution, our EM map is better than the average of the maps deposited in recent years (average in 2018: 6.6 Å). We repeated our structure determination approach using maps truncated to either 6 or 8 Å in the refinement step. (Note that in steps 1 and 2, our approach is anyhow based on a 8 Å map only.) Figure 4e, f shows the refined structures with these two lower-resolution maps, which still have a decent accuracy, with backbone RMSD values to the crystal structure of 1.8 and 2.6 Å, respectively (calculated over Cα). We additionally explored a refinement with phenix.real_space_refine^29^ to refine these structures furthermore, and obtained an even slightly higher structure accuracy (see Table 1, Supplementary Table 6, and Methods). These results highlight that the combined EM/NMR approach is able to provide near-atomic-resolution structures even in cases where the resolution is worse than the present-day average values, and for which de novo structure determination is currently far out of reach using EM methods only.

## Discussion

The recent “resolution revolution” in cryo-EM is progressively changing the way how the structures of biomolecular assemblies are determined, but currently de novo structure determination remains reserved to a minority of EM data sets. Even with the comparably favorable 4.1 Å resolution of our TET2 EM map we were unable to trace the polypeptide chain de novo using currently available tools (see Supplementary Fig. 7). The integration of complementary data from different techniques holds great promise for obtaining atomic-resolution structures even when any single method provides too sparse data. The present work is among the few examples of such an integration of EM and NMR data, and allowed us to obtain an atomic-resolution structure of a protein with a size well beyond typical NMR studies. Compared to these previous structures determined from NMR/EM data^12–14^, TET2 has a more than four times larger monomer size, being larger than any protein for which near-complete MAS NMR resonance assignments have been reported so far. Both the NMR and EM analyses are facilitated by the inherent symmetry of TET2, as the NMR spectral complexity is the one of a 39 kDa protein, not a 468 kDa particle. The extension to larger systems and systems without symmetry will come with challenges in terms of NMR resolution and sensitivity, because the sensitivity is spread over a larger number of peaks. Nonetheless, we believe that ongoing progress in NMR will rapidly push the frontiers: this study has used almost exclusively moderate magnetic field (primarily 600 MHz NMR), far below the current highest magnetic field available for biological NMR studies (1 GHz). Furthermore, the advent of cryogenic MAS NMR probes will further speed up data collection of high-dimensional spectra, which we have shown here to be crucial.

Our approach uses features that can be identified in a straightforward manner even in medium (8–10 Å) resolution EM maps, and in an automatic unsupervised manner, most commonly α-helices. Although not exploited here, also some β-sheet regions and bulky side chains could be readily identified in the EM maps (cf. Supplementary Fig. 9A) and exploited similarly as the α-helices used here. The inclusion of only very few such real-space restraints—in our case only five points along the sequence—turns out to be very powerful in driving the structure calculation towards the correct fold. Specific isotope-labeling schemes, in particular deuteration and methyl-labeling, were crucial as they allowed the unambiguous assignment of hundreds of solution- and solid-state NMR-distance restraints between residues distant in primary sequence, thereby enabling to use the NMR-score to identify the correct helix-to-density assignment, which is the first step to guide the structure towards the correct topology. A remarkable result of this study is the finding that even an EM map at 8 Å resolution allowed us to determine the fold accurately, whereas the de novo modeling even with a 4.1 Å resolution map failed. While the collection of a larger data set from purified particles may allow solving structures from EM alone, our approach may become particularly interesting for cryo electron tomography, where the collection of large data sets is challenging, and the resolution is often limited to ca. 10 Å.

Data from other sources can be readily included into this integrated approach. The distance restraints obtained here from solution-state and MAS NMR may be complemented or replaced by distance information obtained from co-evolution data^30–33^, cross-linking experiments^34^, Förster resonance energy transfer^35^, DEER electron paramagnetic resonance^36–38^, or NMR-detected paramagnetic relaxation enhancement (PRE) data^39^. The secondary structure information obtained here from NMR chemical-shift assignments may be complemented with prediction algorithms in case of missing NMR assignments. Integrating such diverse data in structure determination will likely play an important role in structural studies of challenging biological assemblies forming oligomeric structures, such as capsids, tubes/needles/fibers, i.e., structures that generally are very difficult to study by crystallography. Current developments in our laboratory and by other groups^40^ aim at assessing quantitatively how such data will expand the capability to determine structures with such integrated approaches.

## Methods

### Sample preparation

TET2 from *P. horikoshii* (UniProt entry O59196) was obtained by overexpression in *Escherichia* *coli* BL21(DE3) (Novagen) cells with suitable isotope-labeled M9 minima, supplemented with ^15^NH_4_Cl in all cases as sole nitrogen source. Depending on the desired labeling scheme (outlined below), suitably labeled D-glucose (uniformly ^13^C_6_-labeled or 1,2,3,4,5,6,6-^2^H_7_,^13^C_6_-labeled or 1,2,3,4,5,6,6-^2^H_7_-labeled or unlabeled) was used as carbon source, and for the case of samples with amino-acid specific labeling, isotope-labeled amino acids or ketoacid precursors were added before induction with isopropyl β-D-1-thiogalactopyranoside (IPTG, 1 mM in final culture).

For MAS NMR, six different samples were used in this study with different labeling. Sample 1: u-[^13^C,^15^N]. Sample 2: u-[^15^N],Gly,Tyr,Phe,Arg-[^13^C]. Sample 3: u-[^15^N],Ile,Leu,Val-[^13^C]. Sample 4: u-[^15^N]-Leu,Lys,Pro-[^13^C]. Sample 5: u-[^2^H,^13^C,^15^N]. Sample 6: u-[^2^H,^15^N], Ile^*δ*1^,Leu^*δ*2^,Val^*γ*2^-[^13^CHD_2_], i.e., ^13^CHD_2_ methyl groups at Ile-*δ*^1^, Val-*γ*^proS^ and Leu-*δ*^proS^ positions in an otherwise perdeuterated background (the pro-S methyl is the one denoted as *γ*2 in valine and as *δ*2 in leucine).

For sample 1, ^13^C_6_-labeled glucose was used. For samples 2–4, unlabeled D-glucose was used. For sample 3 ^13^C_4_-2-ketobutyrate (60 mg/L of culture, NMR-Bio) was added 20 min prior to induction and ^13^C_5_-acetolactate (300 mg/L, NMR-Bio) was added 1 h prior to induction. The resulting Ile residues have ^13^C labels at the C^α^, CO, C^γ1^, and C^δ1^ sites; the leucines bear ^13^C at the β, γ, and δ sites; the valines are uniformly ^13^C labeled^21^. For samples 2 and 4, the u-[^13^C,^15^N]-labeled amino acids were added 1 h prior to induction^41^. For sample 5, D_2_O-based M9 medium was used, with ^2^H_7_,^13^C_6_-labeled glucose. For sample 6, Ile^δ1^,Leu^δ2^,Val^γ2^ sites were labeled by addition of 2-ketobutyrate (60 mg/L; 20 min prior to induction) and acetolactate (200 mg/L; 1 h prior to induction), with ^13^CHD_2_-groups at the relevant positions^21,42^. For the production of the deuterated, methyl-labeled samples, cells were progressively adapted, in three stages, over 24 h, to M9/D_2_O media containing 1 g/L ^15^ND_4_Cl and 2 g/L ^2^H_7_ glucose (Isotec). In the final culture, the bacteria were grown at 37 °C in M9 media prepared with 99.85% D_2_O (Eurisotop).

Samples 1–4 were used for resonance assignment with ^13^C-detected MAS NMR experiments (see Supplementary Table 2). Sample 5 was used for ^1^H-detected MAS NMR assignment experiments, listed in Supplementary Table 4. Sample 6 was used to collect ^1^H–^1^H distance restraints between methyl and amide groups by RFDR MAS NMR (see Supplementary Table 5). Sample 4 was furthermore used to collect ^13^C–^13^C distance restraints.

For solution-state NMR NOESY experiments, a uniformly deuterated sample with ^13^CH_3_ groups at Ile-δ^1^ and Val-γ^proS^ sites was used (sample 7)^18^. We refer to this labeling as u-[^2^H], u-[^15^N], Ile^*δ*1^,Val^*γ*2^-[^13^CH_3_]. This sample was used for collecting methyl-NOESY spectra, listed in Supplementary Table 5. Three additional samples were used for the identification of intermolecular contacts, labeled either with u-[^2^H,^15^N],Ile^δ1^,Thr^γ2^,Val^proS^-[^13^CH_3_] (sample 8), or u-[^2^H,^15^N], ${\mathrm{Met}}^\epsilon$, Ala^β^, Val^proR^-[^13^CH_3_] (sample 9), and a sample in which these two types of subunits were mixed at acidic pH and re-assembled at neutral pH following previously published protocol (sample 10)^43^, as described in Supplementary Fig. 4E–G. In the present work, the detection of inter-subunit contacts, however, turned out to be not critical, and these three samples and the according NOESY data sets were used only to exclude eight inter-subunit contacts.

Following protein expression, cells were harvested by centrifugation and broken in a microfluidizer (15 kpsi, 3 passes) in 50 mM Tris–HCl/150 mM NaCl buffer. The total protein extract from disrupted cells was heated to 85 °C for 15 min and the lysates cleared by centrifugation (17,000 *g*, 1 h, 4 °C). The supernatant was loaded onto a 6-mL Resource Q column (GE Healthcare) equilibrated in 100 mM NaCl, 20 mM Tris–HCl, pH 7.5. After washing with 3 column volumes, the protein was eluted with a linear salt gradient (0.1–0.35 M NaCl in 20 mM Tris–HCl, pH 7.5), followed by a size exclusion chromatography step (HiLoad 16/60 Superdex 200, GE Healthcare). Sample purity was verified by SDS-PAGE, and protein-containing fractions were pooled and concentrated. NMR samples used for the solution-state NMR NOESY experiment were at a protein subunit concentration of 1 mM (i.e., a concentration of dodecameric particles of 83 μM) in a D_2_O-based buffer (20 mM Tris, 20 mM NaCl, pH 7.4) in a 5 mm Shigemi tube. Samples for MAS NMR were prepared by concentrating TET2 to 10 mg/mL in H_2_O-based buffer (20 mM Tris, 20 mM NaCl, pH 7.4) and then adding 2-Methyl-2,4-pentanediol (MPD), a commonly used crystallization agent, in a 1:1 (v/v) ratio. A white precipitate (possibly of nanocrystalline nature) appeared immediately after mixing of the solutions. The samples were filled into either 1.3 mm (Bruker), 1.6 mm (Agilent), 3.2 mm (Agilent), or 3.2 mm (Bruker) rotors, using an ultracentrifuge filling device adapted for a Beckman SW32 ultracentrifuge. Typically, samples were filled for ca. 1 h at ca. 50,000 × *g*. Rotors were glued with two-component epoxy glue to avoid loss of solvent.

A sample of u-[^2^H,^13^C,^15^N]-labeled TET2 was used for cryo-electron microscopy. The isotope labeling, although unnecessary for cryo-EM, was useful to record ssNMR spectra with the same sample, in parallel to cryo-EM as a sample quality control. Three and a half microliters of sample were applied to 2:1 glow discharged Quantifoil holey carbon grids (Quantifoil Micro Tools GmbH, Germany) and the grids were frozen in liquid ethane with a Vitrobot Mark IV (FEI, the Netherlands).

### NMR spectroscopy

All solution-NMR experiments were performed at a temperature of 50 °C, either with a Varian INOVA 800 MHz spectrometer (for the H–H–C NOESY) or a 950 MHz Bruker Avance 3 spectrometer, both equipped with cryogenically cooled probes. MAS NMR experiments were performed at an effective sample temperature of ca. 28 °C, measured from the bulk water resonance and the resonance of MPD at 4.1 ppm. MAS NMR assignment experiments were recorded either on an Agilent 600 MHz VNMRS spectrometer equipped with a triple-resonance 3.2 mm probe (for ^13^C-detected experiments) or a 1.6 mm Fast-MAS probe (for all reported ^1^H-detected experiments at a MAS frequency below 40 kHz), or a Bruker 1000 MHz Avance spectrometer equipped with a 3.2 mm probe (only one 3D experiment, see Supplementary Table 2). All additional ^1^H-detected experiments at MAS frequencies >50 kHz were recorded with a Bruker 600 MHz Avance 3 spectrometer equipped with a 1.3 mm MAS probe tuned to ^1^H, ^2^H, ^13^C and ^15^N frequencies. Acquisition parameters of all experiments are reported in Supplementary Tables 2–5.

Assignment of NMR resonances by MAS NMR was performed with a suite of 3D and 4D experiments with either ^13^C detection or ^1^H detection, listed in Supplementary Tables 2–4.

Inspection of the spectra and manual peak picking was performed with the CCPNMR software^44^. Near-complete resonance assignments were achieved by manual analysis. Hereby, the two 4D spectra, CONCACB and CANCOCX, were crucial to unambiguously identify neighboring spin systems. They share three frequencies (CA, N, CO), and the combination of the two spectra thus allows unambiguously connecting five or six frequencies. Comparing patterns in these spectra, as well as in 3D NCACX, NCOCX, and NCACB spectra allowed the sequential connection of such 5-tuples of frequencies. Assignments of the side chains were obtained from the three-dimensional NCACX, NCOCX, and CCC experiments. The assignment of all reported heavy atoms was achieved from ^13^C-detected experiments only. The additional ^1^H-detected experiments were used to assign the amide-^1^H frequency.

Following the manual assignment we investigated the ability to obtain fully automatic assignments. Peaks in all spectra listed in Supplementary Tables 2–4 were picked manually (numbers of picked peaks are listed in Supplementary Tables 2–4), and used as input for the automated assignment algorithm FLYA^19^, implemented in CYANA version 3.97^22^. The output of the automatic assignment procedure is shown in Supplementary Fig. 3.

This suite of experiments allowed the assignment of the heavy atoms (^13^C, ^15^N) and amide ^1^H. The additional assignment of methyl groups was achieved by a mutagenesis-based solution-NMR assignment approach, reported previously for Ile^17^ and Val^18^. These assignments were confirmed and extended to Leu by the ^13^C assignments obtained by MAS NMR.

In cases where several methyl groups have the same ^13^C frequency, this information was insufficient by itself. Thus, we furthermore used the information from the RFDR MAS NMR experiment, in particular cross-peaks from the methyl group to the amide backbone site. All resonance assignments are reported in the BioMagResBank (BMRB ID 27211).

Supplementary Table 5 summarizes all acquisition parameters of solution-state and MAS NMR experiments used for determining distance restraints. Two types of solution-state NMR NOESY spectra were collected. In a first approach, a u-[^2^H, ^15^N], Ile-[^13^CH_3_]^δ1^, Val-[^13^CH_3_]^pro-S^ labeled sample was used to collected NOESY distance restraints with a 3D H–H–C edited experiment^18^. In order to exclude inter-subunit contacts, which might induce errors in single subunit structure calculations, we additionally measured two 3D H–C–C NOESY experiments: one on a sample in which different subunits were labeled differently (sample 10; see Supplementary Fig. 4E) and two samples where all subunits were labeled identically (sample 8). Eight inter-subunit contacts detected in this experiment were excluded from the calculation.

MAS NMR distance restraints were obtained through a 3D ^1^H–^15^N/^13^C–^15^N/^13^C time-shared RFDR experiment and a 2D ^13^C-^13^DARR experiment (see Supplementary Table 5 and Supplementary Fig. 4).

### Cryo-electron microscopy

The sample was observed with a FEI Polara at 300 kV. Movies were recorded manually on a K2 summit direct detector (Gatan Inc., USA) in super resolution counting mode at a nominal magnification of 20,000 (0.97 Å/pixel at the camera level) for a total exposure time of 4 s and 100 ms per frame resulting in movies of 40 frames with a total dose of ~40 e-/Å^2^. Ninety movies were manually recorded with Digital Micrograph (Gatan Inc., USA). Movies were first motion corrected with Digital Micrograph (Gatan Inc., USA) resulting in micrographs like the one on Supplementary Fig. 6. 1643 particles were picked semi-automatically with boxer from the EMAN suite^45^ and 2D classified in 16 classes in RELION 1.4^46^. The best-defined 2D class averages were used to autopick particles in all the micrographs with RELION resulting in a total of 30407 particles. CTF estimation with CTFFIND3^47^, particle extraction in boxes of 200 × 200 pixels and preprocessing were done in RELION as well. The data set was first cleaned by 2D classification leaving 27130 particles. From the best 2D classes, a low resolution *ab initio* 3D model was generated using the RIco server^48^ imposing tetrahedral symmetry. The latter was then used as a starting model to refine all particle orientations in RELION (with tetrahedral symmetry imposed). Further 3D classifications were attempted but did not result in any improvement of the structure. The final 3D reconstruction has a resolution of 4.1 Å at FSC = 0.143. Supplementary Fig. 10A shows that the resolution of the obtained EM map is isotropic as estimated by local resolution (3D FSC) measurements^49^. It also shows a uniform distribution of views with some accumulation of views along the symmetry axes as it is expected for objects with flat faces. The FSC plots in Supplementary Fig. 10B show the EM map-to-model agreement for the various resolutions for both the X-ray structure (PDBid: 1y0r) and the atomic model resulting from proposed procedure (PDBid: 6f3k).

### Structure calculation

The protocol described in this work employed standard programs routinely used in NMR structure determination and EM fitting, namely CYANA (version 3.97)^22^, XPLOR-NIH (version 2.44.8)^27^, iModFit (version 1.44)^26^, helixhunter2^25,50^, Phenix as well as in-house written scripts in python language (available from the authors upon request).

Step 1a consists of the detection and assignment of α-helices. The automatic detection of the α-helix and β-sheet regions from the cryo-EM map was done with the 8 Å resolution map using helixhunter2^25,50^ and gorgon^51^. helixhunter1, was able to detect only the five longer alpha helices, while both helixhunter2 and gorgon added two shorter α-helices and the latter also detected β-sheet regions that are shown in Supplementary Fig. 8A. We used only the five longest α-helices (and their 11 symmetry mates across the dodecamer), denoted A–E in Fig. 1g and Supplementary Table 1, for generating structure restraints used in step 1 of the protocol.

While not strictly necessary in this protocol, for reasons of computational efficiency it is helpful to be able to identify which α-helices belong to a given TET2 monomer, as it allows reducing the number of helix-to-density assignments if only helical densities within one subunit need to be considered. We clustered these 12 × 5 helical densities into groups corresponding to individual subunits by an automatic approach based on the “density-based spatial clustering of application with noise (DBSCAN)” algorithm that was implemented in the Sklearn python package (https://pypi.org/project/sklearn/#history), and the outcome is shown in Supplementary Fig. 8. In essence, the underlying assumption is that the α-helices that are closest to each other belong to the same subunit. For the case of the five helices we considered in TET2, this assumption turns out to be correct. Note, however, that our approach does not rely on the validity of the assumption that the closest helices belong to one subunit; any other combinations, including (erroneous) assignments of one chain to densities belonging to different subunits can equally be considered in the CYANA calculations. We have tested a number of such erroneous helix-to-density assignments, and all these calculations yielded very high CYANA scores and could be discarded on this basis. Thus, erroneous assignment of one chain to different subunits is detected through lack of convergence along the protocol.

Supplementary Table 1 shows the helix-to-density assignment possibilities which we have taken into account in step 1, based on the lengths of the α-helices. In addition to considering the helix lengths, all the physically meaningless assignments in which the same α-helix would be placed at two or more locations at the same time are filtered out. The 40 possible assignments, listed in Supplementary Table 1, were used to generate lists of residue numbers (the centers of the NMR-detected α-helices) assigned to coordinates in space (the centers of the five α-helical densities). These inter-helix distance restraints were used in the subsequent CYANA calculations.

Step 1b consists of CYANA structure calculations and ranking of the helix assignments. Forty CYANA calculations (version 3.97)^22^ were performed, using as restraints the unambiguous distance restraints, backbone dihedral-angle restraints and the restraints of helix-centers to helical-density centers. In all CYANA calculations performed in this study, only one chain (353 residues) was used, and the dodecamer was built from 12 chains only in step 3. Because CYANA works in dihedral-angle space in a protein-internal frame, rather than in real space, the implementation of the real-space helix restraints required translating the absolute-space information into relative-space information. In practice, distance restraints were applied between all atoms that were to be fixed in real space, e.g., between the five helix-centers in step 1. For practical purposes, this method results in fixing the relative orientation and distance of these points, but leaves translational and rotational freedom to the whole protein, which is inconsequential for the resulting structure.

For each helix-to-density assignment, one-thousand conformers were computed using the standard simulating annealing protocol in CYANA with 4000 torsion-angle dynamics steps per conformer. Typical computation times for generating these 1000 conformations was ca. 8.3 min on an Intel 8-core desktop computer. Finally, the 10 conformers with the lowest final target energy function were retained, and their energy was used as one criterion for ranking of the assignments. This CYANA score is plotted as one of the axes in Fig. 2b. Note that out of the 40 CYANA runs, 20 were stopped due to divergent target function energy, showing that these helix-to-density assignments were incompatible with the other NMR data. The 20 remaining calculations are shown in Fig. 2b.

The second criterion for selecting the correct assignment was the goodness of the fit between the EM map and the structure, evaluated only for the five helices considered in a given helix-to-density assignment. Basically, the idea is to evaluate whether all residues of the helix reside within the experimental map; incorrect helix-to-density assignment would lead to a distorted structure and therefore the helices would not fit in the EM map.

To implement this criterion in practice, electron-density maps were generated in silico from the lowest-energy CYANA model, using the backbone N, Cα, C, and O atoms of each of the residues in the five helices which were used in the given helix-to-density assignment (as identified by TALOS-N), using the module “molmap” in UCSF Chimera^52^. We compared these in silico maps to an experimental map, which comprised only the five α-helices considered (colored and labeled A–E in Fig. 1g, map truncated to 8 Å resolution). The module “measure correlation” in UCSF chimera was used to compute the correlation between the in silico maps (of each residue) and this experimental “helix-density” map. This correlation coefficient, obtained residue by residue, reflects the goodness of fit to the map. Examples are shown in Supplementary Fig. 9. As a global measure, we computed the percentage of residues that had a good overlap (>0.7, in which 1.0 and 0.0 means good and bad overlap between the maps, respectively). This percentage is shown in Fig. 2b as vertical axis.

In step 2, an iterative assignment step, we performed rounds of flexible fitting using a common tool used in EM (iModFit^26^) and an NMR-type structure calculation with CYANA. For the latter we used the information about the match to the EM map, similarly as the five helix centers were restrained in step 1. This procedure is described as follows.

The structure resulting from step 1 was taken as a seed to perform a normal-mode based flexible fitting into the density map with the software iModFit v1.44^26^. After this procedure, we identified those protein regions which resided well within the experimental Coulombic potential, in order to be able to fix those residues in the following CYANA calculations. This identification how well the residues correspond to intensity in the experimental Coulombic potential map was done in a similar manner as the goodness-of-fit criterion described in the preceding paragraph, using UCSF chimera modules molmap (allowing to generate density maps from a PDB file) and “measure correlation” (allowing the computation with an experimental map), as follows. We created an in silico map for each residue (backbone N, Cα, C, O only) and computed its correlation to the experimental EM map, truncated to 8 Å resolution, using the full map (rather than only the helices used above). Supplementary Figure 9 shows this overlap score for each residue, as well as graphical examples. Residues with a good overlap score to the map (arbitrarily set as >0.7), were restrained in the following CYANA structure calculation. To fix the atoms in real space using CYANA, even though CYANA operates in internal dihedral-angle space, the same procedure was applied as in step 1, i.e., the atoms belong to the backbone were constrained through distance restraints to all those other atoms that were also fixed in real space (using a tolerance of ±0.5 Å). Note that in practice it is of no importance where in absolute space the structure is placed, so long as the EM information is retained through these relative restraints. This list of inter-atomic distances was generated using an in-house written python script, and was used in the same manner as the NMR distance restraints, using a tolerance of 0.5 Å.

NMR distance restraints with ambiguous assignments, i.e., resulting from cross-peaks with frequencies that could be assigned to more than one atom-pair, can be disambiguated with the help of the intermediate structure obtained after the flexible fitting. Briefly, for cases with multiple possible assignments (based on NMR chemical-shift positions) we measured the corresponding distances in the intermediate structures for all those atom pairs that were possible assignment candidates based on their frequency. In cases where out of the possible candidates only one atom pair had short a (<8 Å) distance, this assignment was retained in the subsequent CYANA calculation. Note that one may also use the restraints as ambiguous restraints in CYANA, but we found better convergence with this approach.

Furthermore, we also considered that some of the NOESY and RFDR distance restraints may be due to inter-subunit contacts. Introducing these restraints in a calculation that only considers one chain would necessarily lead to distance violations. To identify such cases (of which we had only 8 altogether), 3D HMQC-NOESY-HMQC spectra were recorded on a sample in which differently labeled subunit types were mixed in a 1:1 ratio before reoligomerization in native dodecamer^43^ and which allows recognizing inter-subunit cross-peaks and compared to a uniformly labeled sample (see Supplementary Fig. 4E–G for details). Cross-peaks identified in this way as stemming from inter-subunit contacts were excluded from further analysis.

CYANA calculations in step 2 used this growing list of restraints from the match to the map as well as the increasing list of unambiguous NMR distances along with the NMR dihedral-angle restraints. Only one subunit was used in these calculations; the explicit dodecamer was considered only in step 3.

In the final refinement step, called step 3, all dihedral-angle and distance restraints from NMR as well as the full EM map (in real space), at a resolution of either 4.1, 6 Å (Fig. 4e) or 8 Å (Fig. 4f), were used in a joint calculation of the full dodecamer using XPLOR-NIH^27^ version 2.44.8. Xplor-NIH’s strict symmetry facility^53^ was used to generate subunit coordinates with tetrahedral symmetry from those of a protomer subunit. Initially, protomer coordinates from step 2 were moved as a rigid body to fit the full construct into the EM map, also allowing overall center of mass motion so that the dodecamer could be centered on the map. This starting structure was refined, allowing all protomer degrees of freedom under all restraints using the standard purely repulsive nonbonded energy term. In each refinement calculation 100 structures were calculated differing in random velocities given at the beginning of molecular dynamics simulated annealing.

The spatial restraining effects of the EM map were introduced by performing calculations against progressively higher resolution maps: the 8 Å map, followed by the 6 Å map, and finally the highest-resolution (4.1 Å) map. The fit structure was used as initial coordinates for the first calculation, and the lowest energy calculated structure from each step was utilized as the initial structure for the subsequent calculation. Finally, the outcome of the calculation using the 4.1 Å map was refined in implicit solvent using the EEFx energy function within Xplor-NIH^54^. For generating the structures shown in Fig. 4e, f, this gradual refinement process was stopped before adding the higher-resolution maps (i.e., before adding the 6 or 4.1 Å resolution maps, respectively), in order to evaluate the quality of the structures resolution from lower-resolution EM data.

In a final stage, to locally refine the structure of the Zn_2_ active site, we introduced distance restraints to the zinc ions. The identity of these chelating side chains can be readily predicted from the sequence^55^. At this stage of the protocol, the chelating residues (H68, D182, E213, E235, H323) were already close in space to each other, allowing the inclusion of explicit restraints between these atoms to model the zinc site using generous distances based on those found in crystal structures. Also in this region, restraints were added from residue 323 to residues 92 and 93 based on a subsequent reanalysis of unassigned RFDR cross peaks. The final XPLOR-NIH calculation was performed in implicit solvent with the 4.1 Å resolution map, all NMR restraints and the Zn-site restraints. For the calculation with the lower-resolution maps (6, 8 Å), this refinement in implicit solvent has been omitted. The structure deposited as 6f3k in the PDB corresponds to the result of this refinement.

In addition, we also investigated whether the result can be further improved with Phenix, a commonly used program in crystallography. The lowest-energy structures from the XPLOR-NIH runs, employing EM data at different resolution, were refined against the cryo-EM maps using phenix.real_space_refine^29^. We additionally used as restraints all distance measurements and secondary structure information from NMR. Different refinement schemes were tested, which all included five iterative cycles of rigid-body fit of individual chains and minimization of atomic positions, but also simulated annealing or morphing or local grid search. The results of these refinements are summarized in Supplementary Table 6. We found simulated annealing to be superior to the two other approaches, as well as to not using it. Models obtained by performing step 3 at 6 and 8 Å, respectively, were refined using the same approach, i.e., by five iterative cycles of real-space refinement including rigid-body fit of individual chains, minimization of atomic positions, and simulated annealing. Refinements were carried out both at 6 or 8 Å, and at 4.1 Å, to allow a fair comparison of the low resolution models with that determined by performing step 3 at 4.1 Å. Correlation coefficients between the cryo-EM map and the model were calculated using phenix.real_space_refine, and correspond to the CC_mask_ described by Jiang and Brunger^56^ based on map values inside a mask calculated around the macromolecule. RMSD between the various models and the biological unit derived from the X-ray structure (1y0r) were calculated using the “super” function in PyMOL.

We also attempted to rebuild the TET2 structure de novo, by use of the 4.1 Å cryo-EM map only, using phenix.model_to_map^23^, in the Phenix suite of crystallographic software^24^. The program was able to trace the map, with better success when the symmetry of the particle was calculated from the cryo-EM map rather than inferred from the biological unit derived from the X-ray structure. However, the map quality was too low for Phenix to assign the sequence and thus build a model. We tested a variety of building schemes, the results of some of which are shown in Supplementary Fig. 7.

### Reporting summary

Further information on research design is available in the Nature Research Reporting Summary linked to this article.

## Supplementary Information

Supplementary Information

Peer Review File

Reporting Summary

## Data Availability

The NMR assignment has been deposited in the BioMagResBank (entry 27211). The 4.1 Å EM map has been deposited in the EMDB (entry 4179). The atomic model resulting from the three steps described herein, using in the final step the 4.1 Å EM map, has been deposited in the Protein Data Bank (PDBid: 6F3K). Furthermore, we have used Phenix to further refine the structure, using all NMR and EM data (see Supplementary Table 6). This structure has been deposited in the Protein Data Bank (PDB ID: 6R8N). Other data are available from the corresponding authors upon reasonable request.

## Code availability

The program codes are available from the authors upon request.
