# Supplementary material for: Integrated NMR and cryo-EM atomic-resolution structure determination of a half-megadalton enzyme complex
Source: Nat Commun. 2019 Jun 19;10:2697. doi: 10.1038/s41467-019-10490-9 (PMC6584647; doi:10.1038/s41467-019-10490-9)
Supplement: Supplementary file 1 — Supplementary Information [file 41467_2019_10490_MOESM1_ESM.pdf]

Integrated NMR and cryo-EM atomic-resolution structure determination of a half-megadalton enzyme complex

D.F. Gauto et al

Supplementary Information

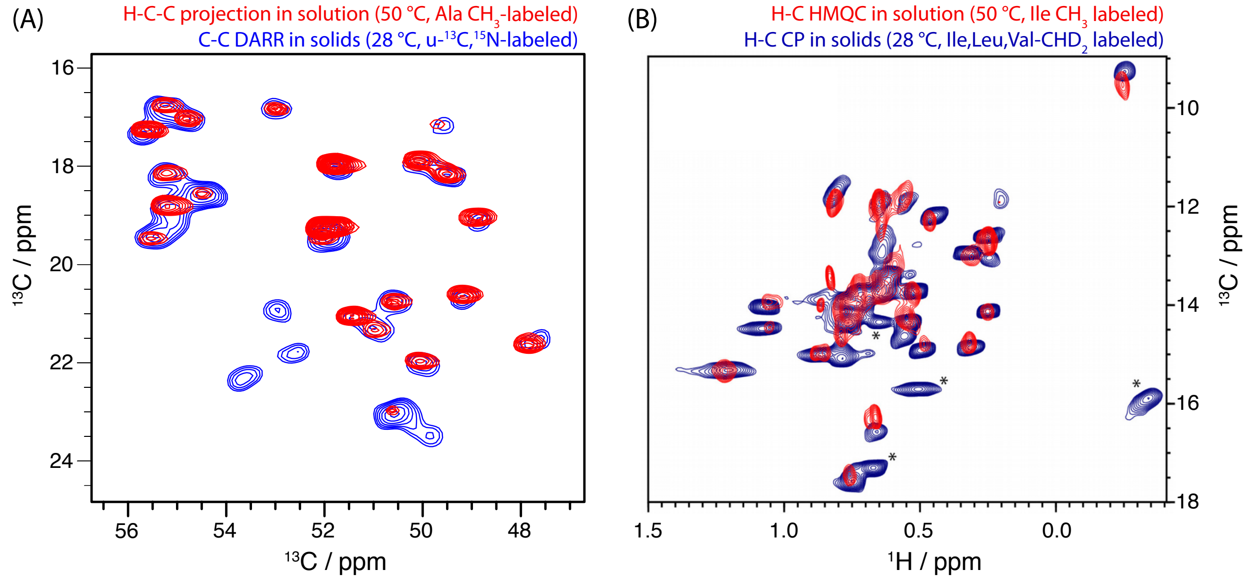

Supplementary Fig. 1: Comparison of solution and MAS NMR spectra of TET2.

(A) Alanine Cα-Cβ correlation spectrum. The solution-state NMR spectrum was collected with a deuterated sample labeled with $^{13}C^{1}H_3$ group and a ^{13}C spin at the Cα position. The spectrum is a ^{1}H detected ^{1}H-^{13}C-^{13}C correlation, projected along the ^{1}H dimension. The MAS NMR spectrum is a DARR experiment, recorded on uniformly ^{13}C,^{15}N labeled sedimented sample, collected at 15 kHz MAS (14.1 T). Note that because of spin relaxation during the transfer steps in the solution-NMR 3D correlation spectrum, several peaks are undetected. The observable peaks match very well the ones in the MAS NMR spectrum.

(B) Comparison of ^{1}H-^{13}C correlation spectra of Ile methyl groups in solution and in an MPD-precipitated TET2 sample. The peaks denoted with an asterisk are resonances from valine sites, which are methyl-labeled in the MAS NMR sample, but not in the solution-NMR sample.

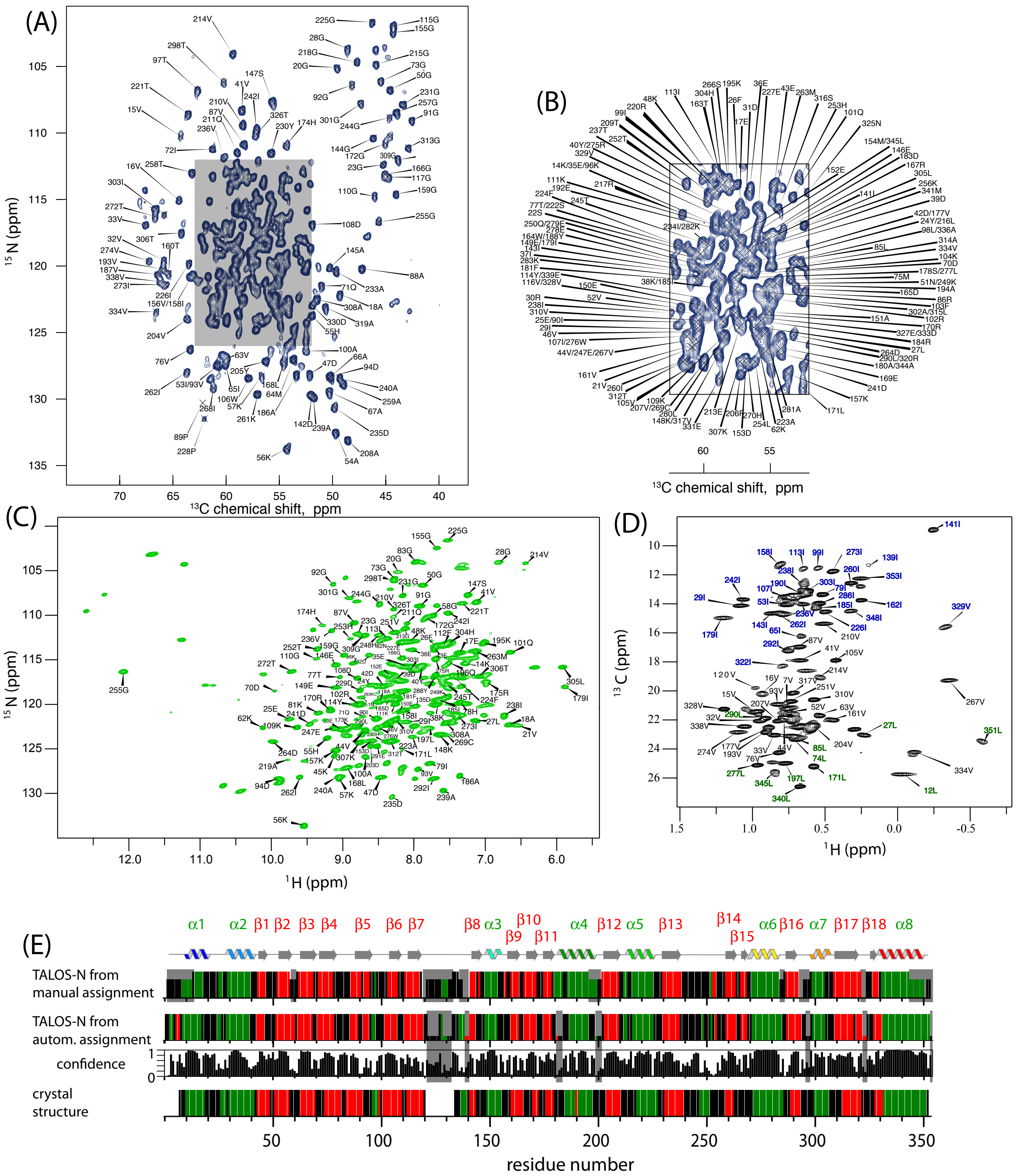
(A)
15N (ppm)
13C chemical shift, ppm
(B)
13C chemical shift, ppm
(C)
15N (ppm)
1H (ppm)
(D)
13C (ppm)
1H (ppm)
(E)
α1 α2 β1 β2 β3 β4 β5 β6 β7 β8 α3 β9 β10 β11 α4 β12 α5 β13 β14 β15 α6 β16 α7 β17 β18 α8
TALOS-N from manual assignment
TALOS-N from autom. assignment
confidence
crystal structure
residue number

Supplementary Fig. 2: Assigned 2D MAS NMR correlation spectra of TET2. (A) NCA correlation spectrum (^{13}C-detected) of uniformly ^{13}C,^{15}N-labeled TET2 recorded at 1000 MHz ^{1}H Larmor frequency, and a zoom of the central part (B).
(C) ^{1}H-detected amide ^{1}H-^{15}N correlation spectrum of uniformly ^{2}H,^{13}C,^{15}N-labeled TET2.
(D) ^{1}H-detected methyl ^{1}H-^{13}C MAS NMR correlation spectrum of u-[^{2}H,^{15}N]-Ile$^{\delta 1}$,Leu^{proS},Val^{proS}-[^{13}CHD$_2$]-labeled TET2 at 600 MHz ^{1}H Larmor frequency (14.1 T). (The pro-S position is also referred to as $\gamma 2$ (in Val) and $\delta 2$ (in Leu).) Ile, Val and Leu methyl groups are annotated in blue, black and green, respectively.
(E) Secondary structure in TET2 derived from MAS NMR chemical shift assignments using the program TALOS-N[1], and comparison to secondary structures in the crystal structure. Loop/coil regions are shown in black, α-helices in green and β-strands in red. Grey bars in the background denote residues for which no backbone assignment was obtained, comprising the long flexible loop from residues 120-133. Note that for residues where no/insufficient chemical-shift assignments are available, TALOS-N uses a database approach to propose secondary structures; these residues are shown as shorter bars in the TALOS-N plot. For comparison, the results from the manual and automatic (FLYA) assignments are shown. The confidence level of the TALOS-N secondary structure predicting is shown for the FLYA data set. The lowest shows the secondary structures as obtained from the crystal structure (PDB entry 1Y0R) and the DSS algorithm, determined with pymol. Missing bars denote residues not modeled in the crystal structure.

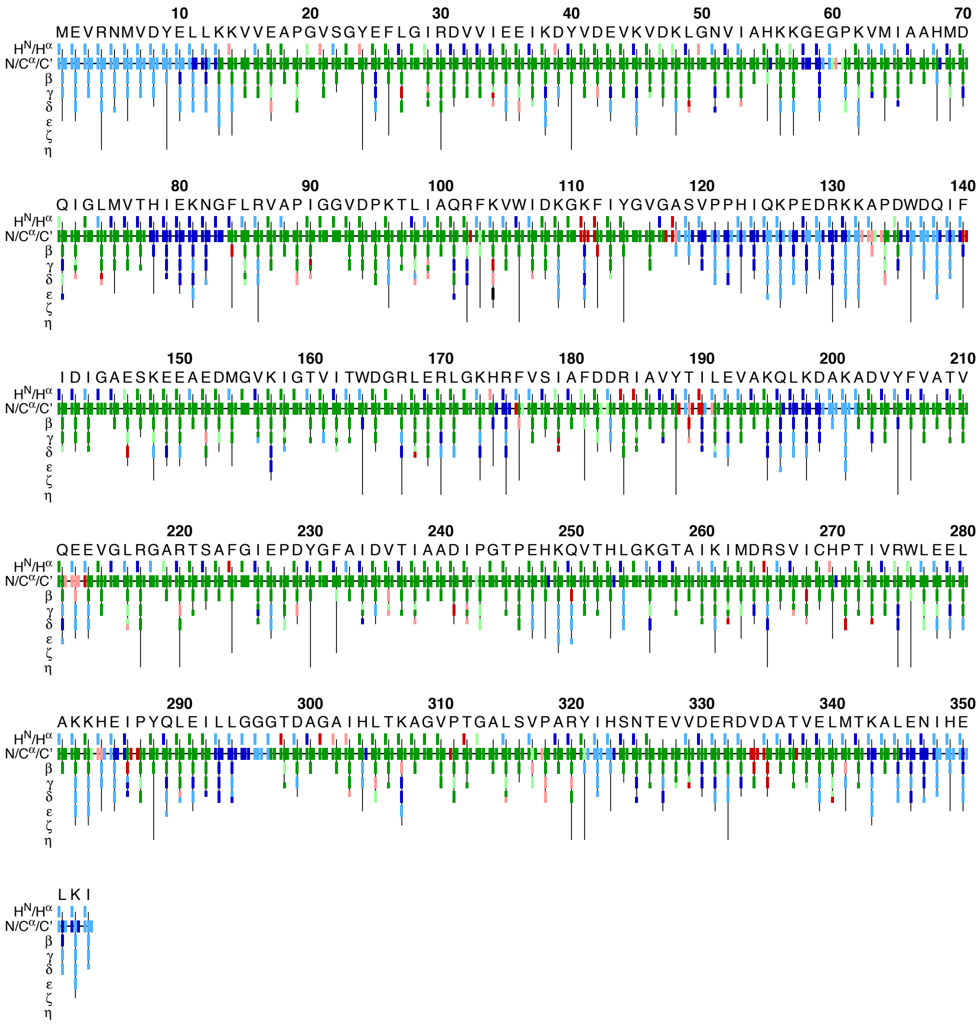

Supplementary Fig. 3: Automatic assignment of MAS NMR spectra of TET2 using the program FLYA.[2] The program used the following spectra for assignment: NCA, NCO, NCOCX, CANCO, NCACX, NCACB, NcoCACB, CONCACB, CANCOCX, NCOCX(GRYF), NCOCX(ILV), NCOCX(LKP), NCACX(GRYF), NCACX(ILV), NCACX(LKP), hCANH, hCONH, hcoCAcoNH, CCCdarrdarr(ILV), CCC, caNCO(GRYF), coNCA(GRYF), coNCA(ILV), coNCA(LKP). The color code reflects the assignment for each atom, and compares it to the manual assignment, as follows. Green: Automatic and manual assignment agree; red: automatic assignment differs from manual assignment; blue: no manual assignment available. Dark colors (green, blue, red) denote atoms for which the FLYA assignment has converged, i.e., the same assignment is found in more than 80% of the 20 independent runs, while light colors denote atoms for which FLYA has not converged, i.e., the results have less confidence. The row labeled H^N/H^α shows for each residue HN on the left. The N/Cα/C' row shows for each residue the N, Cα, and C' assignments from left to right. The rows labeled with β-η show the side-chain assignments for the heavy atoms. In the case of branched side chains, e.g. Val and Leu, the corresponding row is split into an upper part for one branch and a lower part for the other branch.

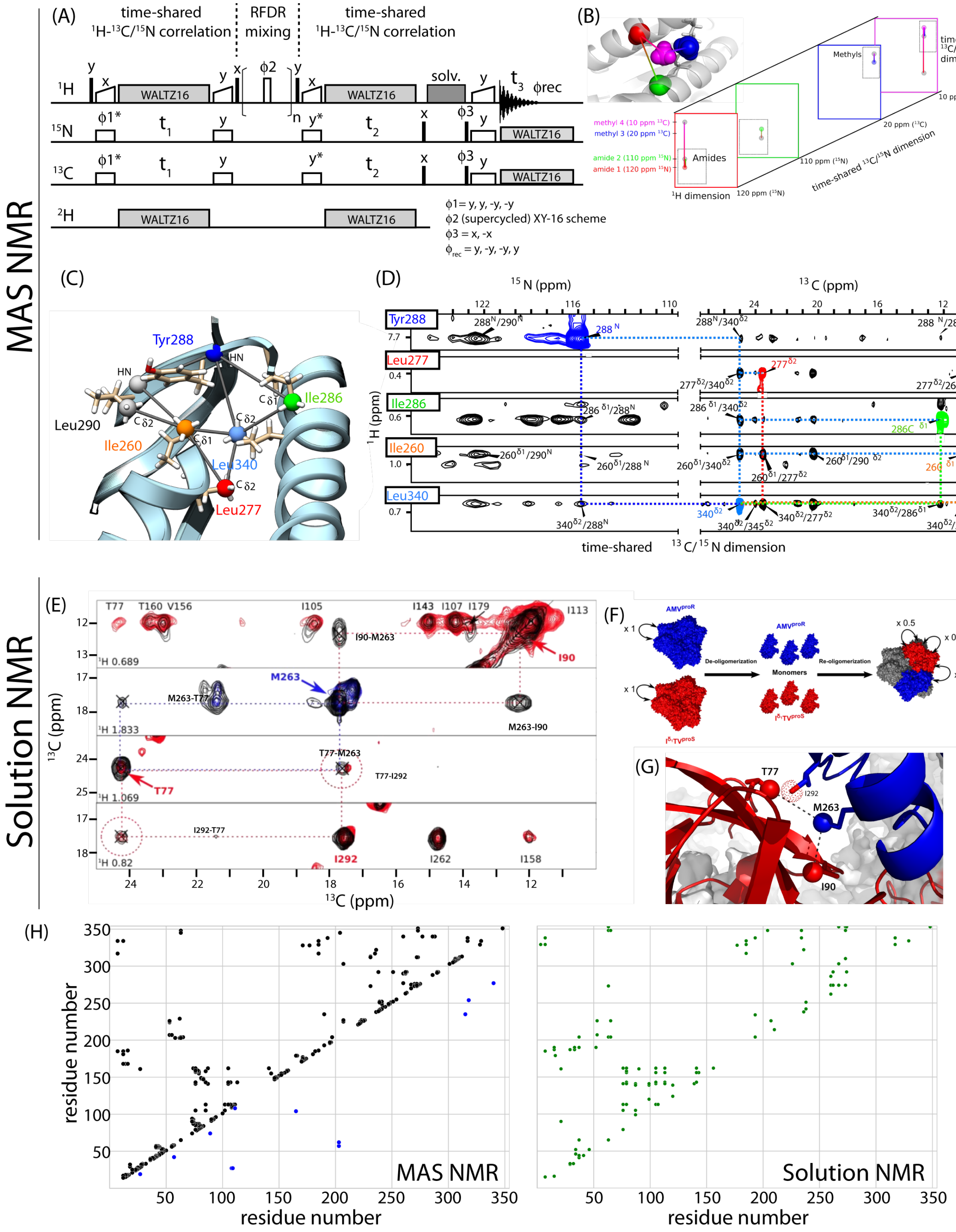
MAS NMR
(A)
time-shared
1H-13C/15N correlation
RFDR mixing
time-shared
1H-13C/15N correlation
WALTZ16
solv.
φrec
φ1=y, y, -y, -y
φ2 (supercycled) XY-16 scheme
φ3 = x, -x
φrec = y, -y, -y, y
(B)
time-shared 13C/15N dimension
Methyls
Amides
1H dimension
10 ppm (13C)
20 ppm (13C)
110 ppm (15N)
120 ppm (15N)
methyl 4 (10 ppm 13C)
methyl 3 (20 ppm 13C)
amide 2 (110 ppm 15N)
amide 1 (120 ppm 15N)
time-shared 13C/15N dimension
(C)
Tyr288
Leu290
Ile260
Ile286
Leu340
Leu277
(D)
15N (ppm)
13C (ppm)
Tyr288
Leu277
Ile286
Ile260
Leu340
1H (ppm)
time-shared 13C/15N dimension
Solution NMR
(E)
T77 T160 V156 I105 I143 I107 I179 I113
I90-M263
I90
M263
M263-T77
M263-I90
T77-M263
T77-I292
T77
I292-T77
I292
I262
I158
13C (ppm)
(F)
AMVproR
De-oligomerization
Monomers
Re-oligomerization
x 0.5
x 0.25
(G)
T77
I292
M263
I90
(H)
residue number
MAS NMR
Solution NMR

Supplementary Fig. 4: Distance restraints from MAS NMR and solution-NMR experiments.
(A) Pulse sequence used for the simultaneous measurement of ^{1}H-^{1}H spatial proximities between amide and methyl moieties under fast-MAS conditions, for application to deuterated, amide-protonated and methyl ^{13}CHD$_2$ labeled samples. Filled and open narrow rectangles denote 90° and 180° pulses, respectively, and wide open rectangles denote cross-polarization steps, applied with a linear ramp on the ^{1}H channel. The RFDR mixing, of a total duration of 5 ms, is comprised of a train of *n* equally-spaced pulses where the centers of two consecutive pulses are separated by the MAS rotor period; the pulse phase is alternated according to the XY16 scheme. The cross-polarization steps simultaneously transfer polarization from amide-^{1}H to ^{15}N and from the methyl-^{1}H to the methyl-^{13}C (out and back). Pulse phases are denoted above the respective symbols. Quadrature detection in the indirect dimensions (t_1, t_2) was performed by varying the phase of the ^{13}C and ^{15}N cross-polarization irradiation prior to the chemical-shift (denoted by an asterisk) according to the States-TPPI scheme. Decoupling of spin-spin couplings during direct and indirection dimensions and the solvent-suppression scheme ("solv.") are denoted by grey rectangles.
(B) Schematic representation of the outcome of this experiment, showing connections between two amide sites, between two methyl sites and between an amide and a methyl site.
(C) Experimental distance restraints from the RFDR experiment shown on part of the TET2 structure.
(D) Excerpts from the 3D RFDR MAS NMR spectrum, showing the cross-peaks supporting the distance restraints shown in (C).
(E) Subunit-specific labeling of TET2 to filter inter-subunit distance information. Example strips from 3D HMQC-NOESY-HMQC spectra recorded on either u-[^{2}H,^{15}N],Ile$^{\delta 1}$,Thr$^{\gamma 2}$,Val^{proS}-[^{13}CH$_3$] (red), u-[^{2}H,^{15}N],Met$^{\epsilon}$,Ala$^{\beta}$,Val^{proR}-[^{13}CH$_3$] (blue) or a sample in which these two differently labeled subunit types were mixed in a 1:1 ratio (black).
(F) Labeling scheme for these spectra. The numbers reflect the relative cross-peak intensities resulting from the stochastic mixing scheme.
(G) Illustration of inter-subunit distance restraints detected between M263 in one subunit and T77 and I90 in another subunit. An additional methyl group from which an intra-subunit contact is observed in the spectra in (C) is shown with a dotted sphere. Note that the inter-subunit distance restraints have not been used directly in this work, but we have only used this information for excluding inter-subunit restraints from the calculations (see Methods). A total of only eight inter-subunit distances has been identified, and this set of experiments might not be crucial in the presented approach.
(H) Residue contact maps showing the restraints from the ^{1}H-^{1}H MAS NMR RFDR experiment connecting amide and methyl sites (black), the ^{13}C DARR experiment on LKP-labeled TET2 (blue), and the solution-state NOESY experiments (green). The eight inter-subunit restraints have been removed from this graph.

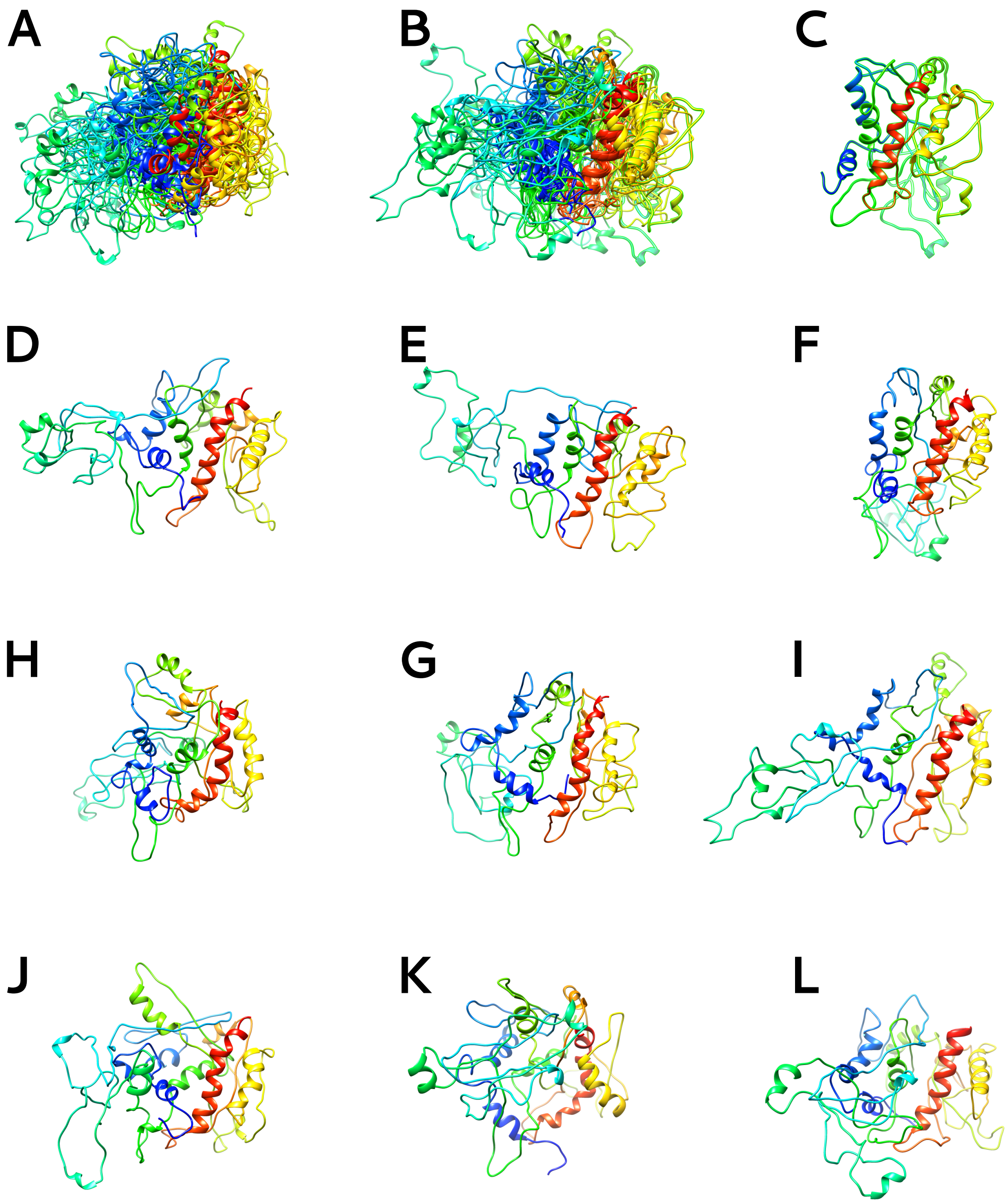

Supplementary Fig. 5: Structure calculation of the monomer of TET2 using only the liquid and solid-state NMR derived restraints. Figure (A) represents the NMR ensemble of 10 structures superimposed on their secondary structure elements (backbone RMSD: 10.5 ± 1.6 Å). Figure (B) shows the same NMR structure ensemble superimposed on the largest helix $\alpha 8$, each structure of this ensemble are displayed individually on figures (C) to (L).

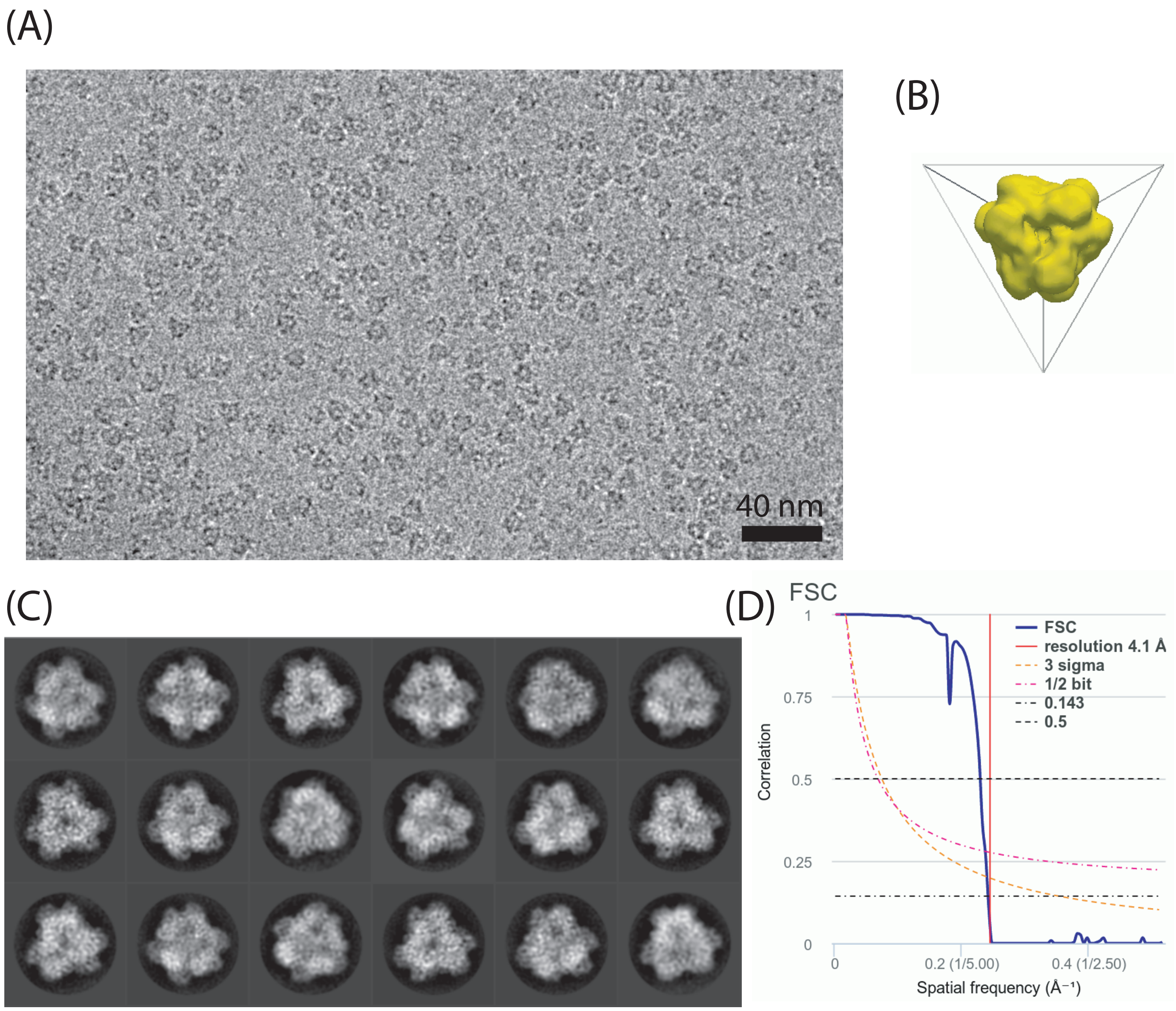

Supplementary Fig. 6: (A) Typical cryo-electron micrograph showing TET particles (darker than the background densities) in random orientations. (B) Used initial low resolution 3D model obtained by imposition of tetrahedral symmetry. (C) Best eighteen 2D class averages obtained from 27,130 particles. (D) Fourier Shell Correlation plot for the presented 3D cryo-EM reconstruction.

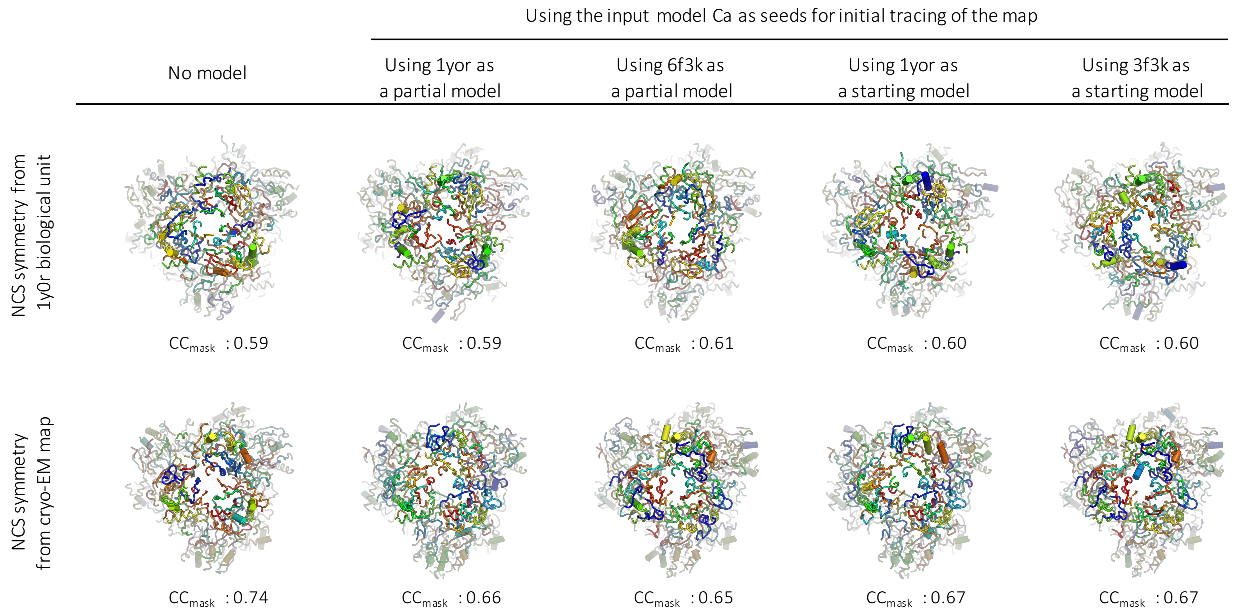

Supplementary Fig. 7: Attempts to determine the structure from the EM map only. Direct rebuilding from the 4.1 Å cryo-EM map fails to produce interpretable structures, regardless of the symmetry that is imposed (or not) for model-building, and of whether (or not) a partial or a starting model is provided. Briefly, we used phenix.map_to_model (10 rebuild cycles) to attempt rebuilding the structure directly from the cryo-EM map (4.1 Å resolution) using either no prior information (no model), or the X-ray (1y0r) or the NMR+cryo-EM (6f3k, this paper) structures as starting coordinates. We tested inclusion of these models either as ”partial” or ”starting” models, and in all cases, imposed the use of the input-model Cα-atoms as seeds for the initial tracing of the map. Rebuilding was attempted with (upper row) or without (lower row) imposing the known symmetry of the particle. In both case, phenix.map_to_model was able to trace the map, but detection of the NCS symmetry from the map (lower panel), as opposed to inferred from the X-ray pdb structure (upper panel), drastically improved the quality of the model. At the map resolution, however, phenix.model_to_map was not able to interpret the Coulombic potential in terms of secondary structure and/or sequence at least not in 10 rebuild cycles. Arguably, the traced model produced by phenix.map_to_model would have been interpretable by an experienced crystallographer or electron microscopist, but reaching the quality of 6f3k would have been very challenging.

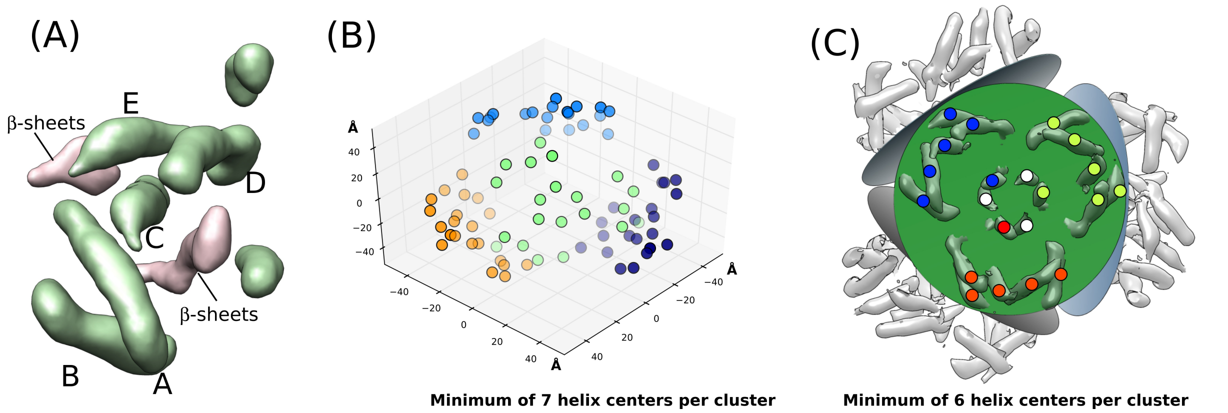

Supplementary Fig. 8: Automatic detection and clustering of secondary structures. (A) Secondary structures detected by *gorgon* [3] from the 8 Å EM map of TET2, zooming on one TET2 subunit. α-helices and β-sheet regions are depicted in green and pink, respectively. Note that in this study we have only used five α-helices, labeled with A to E, and we have not used the information about the location of the β-sheets. Including these regions is straightforward and may prove useful in other applications, with proteins containing primarily β-sheets.

(B,C) Cluster analysis of the α-helices from the EM map, aimed at identifying the α-helices belonging to the same monomer. This automatic distance-based clustering analysis used the python library sklearn.cluster, and needs as input (i) the coordinates of the points in space to be clustered – here, the centers of the 84 automatically detected α-helices –, (ii) the minimum number of elements in each cluster and (iii) the maximum distance between the points belonging to a given cluster. Note that no information about the symmetry or the number of subunits (number of clusters) is provided.

In (B), the minimum number of elements belonging to the same cluster was set to 7. The points distributed in space, representing the centers of the α-helices in the dodecamer, are color-coded according to the group/cluster to which they are assigned. While this clustering does not retrieve the monomer subunits, it detects clusters of 21 points, which correspond to trimers of TET2.

In (C), this analysis was repeated, setting the minimum number of elements per cluster to 6. In this case, the six points assigned to each cluster (shown in blue, red or green) indeed correspond to helix centers of a given subunit. The additional white dots represent the helix centers that could not be assigned to any cluster using the distance-based clustering algorithm. The green plane is shown for facilitating visualization of one trimer within the dodecamer. In Step 1 of our structure calculation protocol we have used the five α-helices within these clusters which are closest in space to each other, omitting the sixth helix.

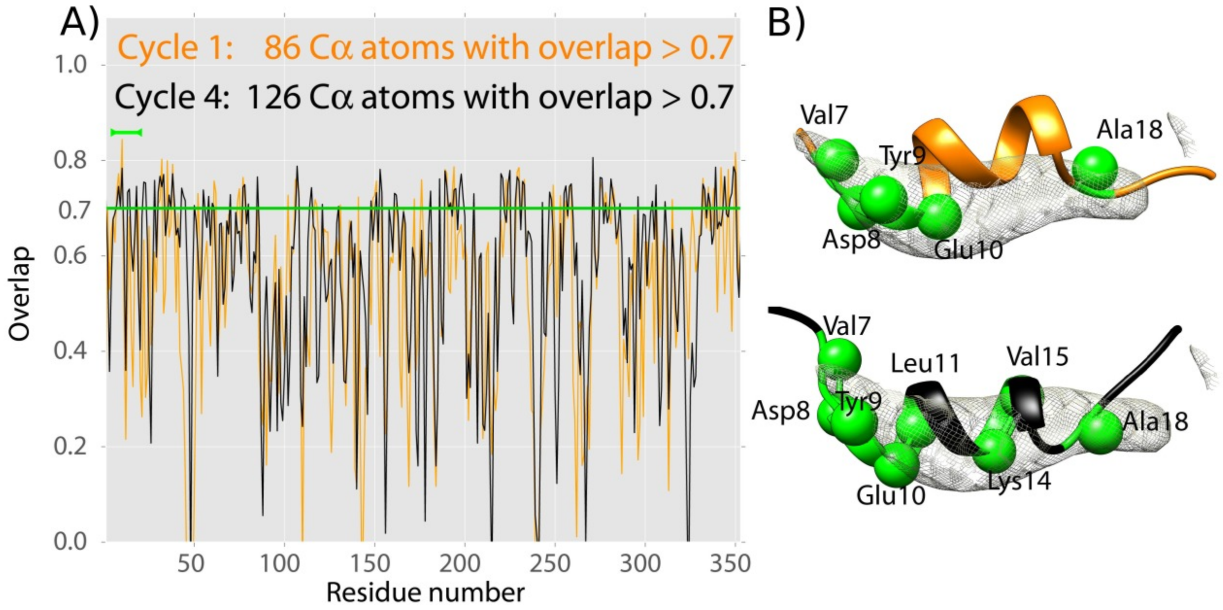

Supplementary Fig. 9: Illustration of the procedure through which, in Step 2 of this approach, residues were restrained to real-space coordinates, based on their overlap with the EM map. Briefly, after flexible fitting with iModFit the structure was compared to the 8 Å EM map to evaluate how well different parts of the chain reside inside the density. To this end, the *molmap* and *measure correlation* modules in UCSF Chimera were used to compute the overlap of an in silico map, constructued from the backbone atoms of a given residue, with the experimental map. The correlation between these two maps, a value ranging from 1 (atom is fully inside density) to 0 (atom outside density) was used as a criterion whether the residue resides within the map or not. If the value was above 0.7 for a given residue, the Cα atom of this residue was then kept in place in the subsequent CYANA calculation by adding restraints from this atom to the other atoms that are fixed in space (using a tolerance of 0.5 Å.)
(A) Residue-wise plot of the overlap score in the first and fourth iteration of Step 2. The green bar on the top left indicates the residues which are shown in (B).
(B) Example of the overlap in the first and fourth iteration. Cα atoms with an overlap score above 0.7 are indicated with green spheres and were constrained in space with a tolerance of $\pm$ 2 Å.

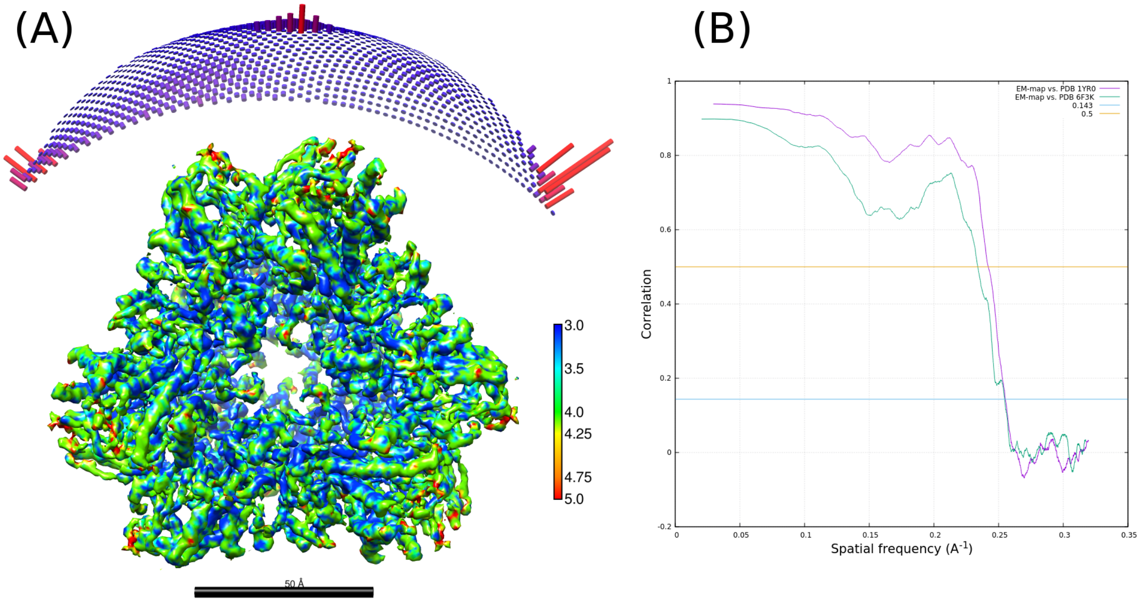

Supplementary Fig. 10: (A) Local resolution of the EM density map colored according to resolution (in Å) estimated by ResMap [4] and histogram of (Euler) views distribution shown as blue-red cylinders. (B) FSC between EM map and atomic models obtained by X-ray crystallography and by the proposed method.

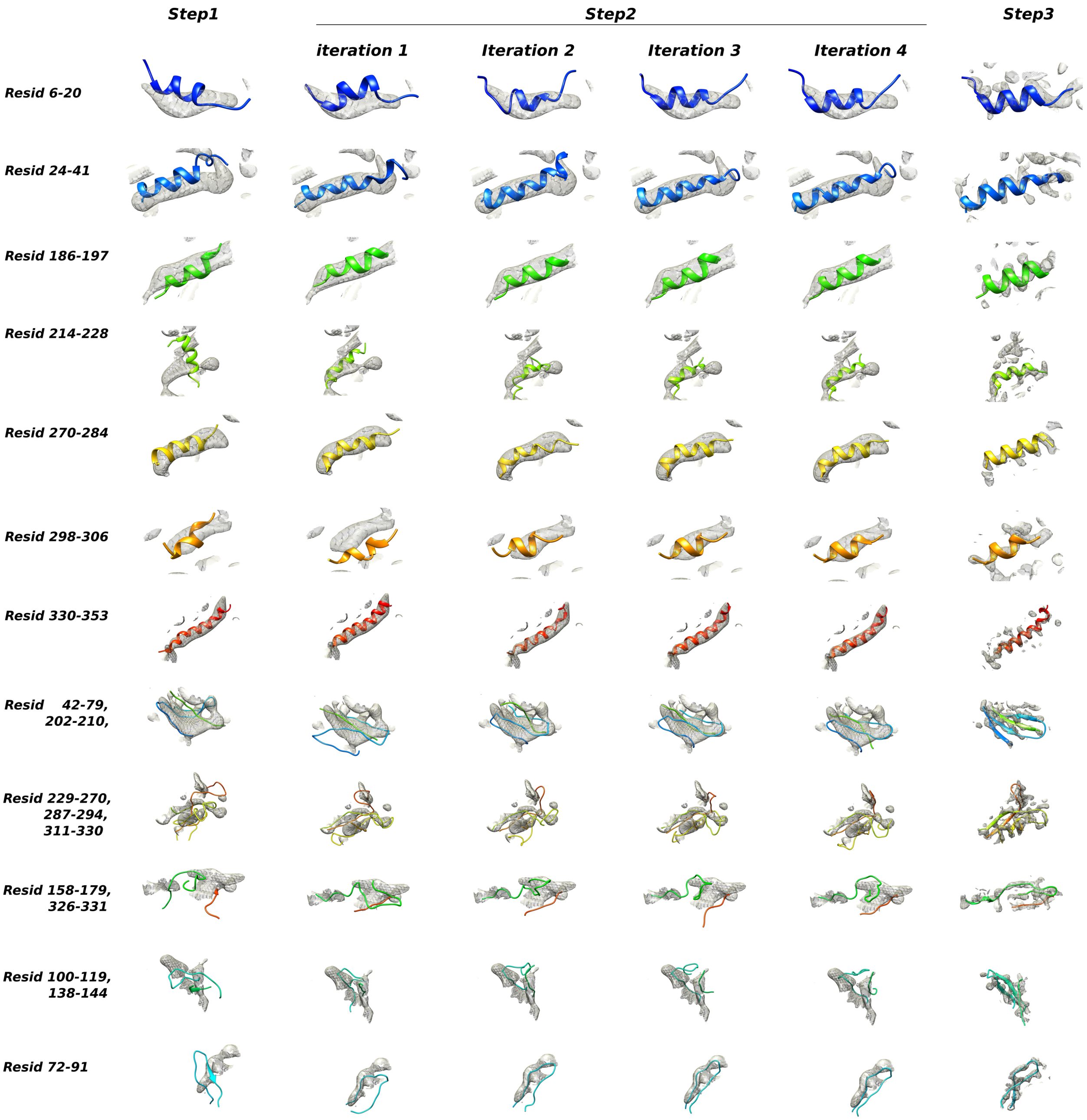

Supplementary Fig. 11: Evolution of the structure of TET2 along the various steps of the integrated NMR/EM structure calculation approach, denoted as Step 1 to 3 in Figure 3. The structures shown correspond to the lowest-energy models generated by CYANA (Steps 1 and 2) or XPLOR-NIH (Step 3).

(A) α helices cryo-EM	Length (cryo-EM)	α helices NMR
A	30.8 Å	α8
B	20.2 Å	α6,α4
C	21.7 Å	α6,α4
D	18.1 Å	α6,α4,α5,α2,α1,α7,α3
E	13.1 Å	α5,α2,α1,α7,α3
(B) Residues placed in centers of the α-helices and estimated lenghts in Å		
α1	Lys 13	13.0 Å
α2	Ile 34	13.0 Å
α3	Ala 151	8.6 Å
α4	Leu 191	15.8 Å
α5	Arg 220	13.0 Å
α6	Leu 277	18.7 Å
α7	Ile 303	8.6 Å
α8	Met 341	31.7 Å
(C) Distance restraints corresponding to the correct helix-to-density assignment		
Met 341 Cα	Leu 277 Cα	10.84 ± 0.50 Å
Met 341 Cα	Leu 191 Cα	10.46 ± 0.50 Å
Met 341 Cα	Ile 34 Cα	20.36 ± 0.50 Å
Met 341 Cα	Lys 13 Cα	19.24 ± 0.50 Å
Leu 277 Cα	Leu 191 Cα	18.30 ± 0.50 Å
Leu 277 Cα	Ile 34 Cα	28.11 ± 0.50 Å
Leu 277 Cα	Lys 13 Cα	28.92 ± 0.50 Å
Leu 191 Cα	Ile 34 Cα	10.43 ± 0.50 Å
Leu 191 Cα	Lys 13 Cα	12.49 ± 0.50 Å
Ile 34 Cα	Lys 13 Cα	12.52 ± 0.50 Å

Supplementary Table 1: *(Preceding page)* Details about the helix-to-density assignments. The number of helix-to-density assignments for eight α-helices detected in the sequence, placed into the seven α-helical densities detected in the EM map (i.e., leaving out one helix in each combination), ignoring the helix polarity, can be calculated as $8! = 40320$. Including the helix polarity increases this number to $8! \times 2^7 \approx 5 \cdot 10^6$. Considering the possibility that the seven densities may not be ascribed unambiguously to a given subunit further increases this number. We solved this latter point (identifying densities of one subunit) by a cluster analysis (see Figure 8) and retaining only the five longest helical densities, which are also closest in space. We explicitly disregarded the helix polarity, by using only the center of the helices in the CYANA calculation, letting the rotation free. The number is furthermore greatly reduced by comparing the lengths of α-helices along the sequence with the helical densities.
(A) This table shows the lengths (middle column) of the helical densities from the EM map (denoted as A-E as shown in Figure 2A, left column), and the α-helices along the sequence (right column, see Figure 2A) that may match the lengths of these densities. Hereby, we have considered that a helix has a length of 1.4 Å per residue. In selecting the possible helix-to-density assignments, we applied a tolerance of $\pm$ 2 amino acids (corresponding to $\pm$ 2.8 Å). As a result, only $1 \times 2 \times 5 \times 4 = 40$ helix-to-density assignments remain that are checked by structure calculations.
(B) Identity of the residues assumed to be in the centers of the α-helices, as obtained from the NMR assignments and TALOS-N. The lengths of the helices in Å was estimated from the length in number of residues, as obtained by TALOS-N, assuming a canonical translation length per residue of ca. 1.45 Å.
(C) Practical implementation of the helix-helix distance restraints, shown here for the correct helix-to-density assignment. These distance restraints were used in CYANA, alongside the other NMR distance restraints and backbone dihedral-angle restraints.

Supplementary Table 2: Acquisition parameters of MAS NMR spectra for resonance assignment of protonated, ^{13}C,^{15}N labeled TET2 in 3.2 mm rotors. CP transfer steps between ^{15}N and ^{13}CA or ^{13}CO used a ^{13}C RF carrier either at the C^{α} (54 ppm) or CO (175 ppm) frequency. The MAS frequency is denoted in kHz. All spectra at 14.1 T were acquired on a Agilent spectrometer; the three spectra (two 2Ds and one 3D) at 23.5 T were recorded on a Bruker Avance spectrometer. The two experiments denoted with ”long CP” used a longer ^{1}H-^{15}N cross-polarization to promote proline signals. The column ”peaks” is the number of detected peaks used for the automatic assignment in FLYA.

Experiment	Dim.<br>B_0/T<br>MAS	Transfer 1	Transfer 2	Transfer 3	Transfer 4	direct dimension<br>spectr. width/kHz<br>points/t_{max}	ind. dim. 1<br>spectr. width/kHz<br>points/t_{max}	ind. dim. 2<br>spectr. width/kHz<br>points/t_{max}	ind. dim. 3<br>spectr. width/kHz<br>points/t_{max}	NS<br>d1/s<br>time/h	# peaks
NCA	2D<br>23.5<br>18	CP H-N, 0.9 ms<br>^{1}H: 60 kHz (ramp)<br>^{15}N: 41 kHz	CP N-CA, 4 ms<br>^{15}N: 12 kHz<br>^{13}C: 6 kHz	-	-	^{13}C<br>75 kHz<br>2020/27 ms	^{15}N<br>3.65 kHz<br>80/21.9 ms	-	-	32<br>1.5 s<br>5.5 h	233
NCO	2D<br>23.5<br>18	CP H-N, 0.9 ms<br>^{1}H: 60 kHz (ramp)<br>^{15}N: 41 kHz	CP N-CA, 4 ms<br>^{15}N: 12 kHz<br>^{13}C: 6 kHz	-	-	^{13}C<br>75 kHz<br>2020/27 ms	^{15}N<br>3.65 kHz<br>80/21.9 ms	-	-	8<br>1.5 s<br>1.5 h	264
NcoCX<br>long CP<br>(for Pro)	2D<br>14.1<br>15	CP H-N, 8 ms<br>^{1}H: 67 kHz (ramp)<br>^{15}N: 51 kHZ	CP NCO, 5.5 ms<br>^{15}N: 11 kHz<br>^{13}C: 3.5 kHz (ramp)	C-C DARR, 130 ms<br>^{1}H RF: 15 kHz	-	^{13}C<br>50 kHz<br>1500/15 ms	^{15}N<br>3 kHz<br>64/13.7 ms	-	-	496<br>1.8 s<br>41 h	118
NcaCX<br>long CP<br>(for Pro)	2D<br>14.1<br>15	CP H-N, 8 ms<br>^{1}H: 67 kHz (ramp)<br>^{15}N: 51 kHZ	CP NCO, 5.5 ms<br>^{15}N: 11 kHz<br>^{13}C: 3.5 kHz (ramp)	C-C DARR, 80 ms<br>^{1}H RF: 15 kHz	-	^{13}C<br>50 kHz<br>1500/15 ms	^{15}N<br>4.6 kHz<br>64/13.7 ms	-	-	496<br>1.8 s<br>39.7 h	91
NCOCX	3D<br>14.1<br>15	CP H-N, 1.1 ms<br>^{1}H: 74 kHz (ramp)<br>^{15}N: 58 kHZ	CP NCO, 5 ms<br>^{15}N: 12 kHz<br>^{13}C: 3.5 kHz (ramp)	C-C DARR, 50 ms<br>^{1}H RF: 15 kHz	-	^{13}C<br>40.3 kHz<br>1612/20 ms	^{15}N<br>3 kHz<br>32/10.3 ms	^{13}CA<br>3.5 kHz<br>32/8.8 ms	-	32<br>1.88 s<br>71 h	1172
NCACX	3D<br>23.5<br>18	CP H-N, 0.9 ms<br>^{1}H: 58 kHz (ramp)<br>^{15}N: 50 kHz	CP N-CA, 4 ms<br>^{15}N: 14 kHz<br>^{13}C: 4 kHz (ramp)	C-C DARR, 80 ms<br>^{1}H RF: 17 kHz	-	^{13}C<br>75 kHz<br>2264/30 ms	^{15}N<br>3.65 kHz<br>70/18.9 ms	^{13}CA<br>9 kHz<br>68/7.4 ms	-	8<br>1.95 s<br>81 h	1574
NCACB	3D<br>14.1<br>15	CP H-N, 1.1 ms<br>^{1}H: 63 kHz (ramp)<br>^{15}N: 46 kHz	CP N-CA, 5 ms<br>^{15}N: 12 kHz<br>^{13}C: 3.5 kHz (ramp)	CA-CB DREAM, 4 ms<br>^{13}C avg RF: 7 kHz<br>tan ramp (62 to 138%)	-	^{13}CO<br>40.3 kHz<br>1612/20 ms	^{15}N<br>2.5 kHz<br>40/15.5 ms	^{13}CA<br>6 kHz<br>76/12.5 ms	-	32<br>1.61 s<br>139 h	596
CANCO	3D<br>14.1<br>15	CP H-C, 0.3 ms<br>^{1}H: 59 kHz (ramp)<br>^{13}C: 44 kHz	CP CA-N, 5.5 ms<br>^{15}N: 11 kHz<br>^{13}C: 4.7 kHz (ramp)	CP N-CO, 5.5 ms<br>^{15}N: 11 kHz<br>^{13}C: 4.7 kHz (ramp)	-	^{13}CO<br>40.3 kHz<br>1612/20 ms	^{15}N<br>2.5 kHz<br>40/15.5 ms	^{13}CA<br>6 kHz<br>76/12.5 ms	-	16<br>2 s<br>110 h	499
NcoCACB	3D<br>14.1<br>15	CP H-N, 1.1 ms<br>^{1}H: 76 kHz (ramp)<br>^{15}N: 60 kHz	CP N-CO, 5 ms<br>^{15}N: 11 kHz<br>^{13}C: 4 kHz (ramp)	C-C DARR, 50 ms<br>^{1}H RF: 15 kHz	CA-CB DREAM, 4 ms<br>^{13}C avg RF: 7 kHz<br>tan ramp (54 to 146%)	$^{13}CACB$<br>50 kHz<br>1800/18 ms	^{15}N<br>2400 Hz<br>30/12.1 ms	^{13}CA<br>10 kHz<br>84/8.3 ms	-	16<br>1.85 s<br>87 h	239
CCC	3D<br>14.1<br>15	CP H-C, 2 ms<br>^{1}H: 75 kHz (ramp)<br>^{13}C: 60 kHz	C-C DREAM, 5 ms<br>^{13}C avg RF: 7 kHz<br>tan ramp (56 to 144%)	C-C DARR, 80 ms<br>^{1}H RF: 15 kHz	-	^{13}C<br>40.3 kHz<br>1210/15 ms	^{13}C<br>13 kHz<br>140/10.7 ms	^{13}C<br>29 kHz<br>260/8.8 ms	-	2<br>1.37 s<br>120 h	704
CONCACB	4D<br>14.1<br>15	CP H-C, 3 ms<br>^{1}H: 77 kHz (ramp)<br>^{13}C: 60 kHz	CP CO-N, 6 ms<br>^{15}N: 11.5 kHz<br>^{13}C: 4 kHz (ramp)	CP N-CA, 5 ms<br>^{15}N: 11.5 kHz<br>^{13}C: 4 kHz (ramp)	CA-CB DREAM, 4 ms<br>^{13}C avg RF: 7 kHz<br>tan ramp (65 to 135%)	^{13}CB<br>50 kHz<br>1600/16 ms	^{13}CO<br>2.3 kHz<br>14/5.6 ms	^{15}N<br>2.5 kHz<br>22/8.4 ms	^{13}CA<br>5.6 kHz<br>34/5.9 ms	4<br>1.7 s<br>162 h	279
CANCOCX	4D<br>14.1<br>15	CP H-C, 0.9 ms<br>^{1}H: 77 kHz (ramp)<br>^{13}C: 60 kHz	CP CA-N, 7 ms<br>^{15}N: 11.5 kHz<br>^{13}C: 4 kHz (ramp)	CP N-CO, 6 ms<br>^{15}N: 11.5 kHz<br>^{13}C: 4 kHz (ramp)	CA-CB DARR, 18 ms<br>^{1}H RF: 15 kHz	^{13}C<br>40.3 kHz<br>1612/20 ms	^{13}CA<br>5.6 kHz<br>27/4.6 ms	^{15}N<br>2.5 kHz<br>26/10 ms	^{13}CO<br>2.3 kHz<br>14/5.6 ms	4<br>1.78 s<br>161 h	699

Supplementary Table 3: Acquisition parameters of MAS NMR spectra for resonance assignment from specifically labeled samples. Spectra were recorded on a 600 MHz Agilent spectrometer in 3.2 mm rotors. Three samples were used, labeled uniformly with ^{15}N by addition of $^{15}NH_4$ to the growth medium, and labeled specifically with the specified amino acids (specified in the table), by addition of these amino acids with u-^{15}N,^{13}C labeling. An exception is the labeling of Leu and Val: here, not the amino acids but the ^{13}C-labeled acetolactate was added[5]; as a consequence, ^{13}C is incorporated at the C^{α} position of Val, but not of Leu, and Leu residues are unobservable in all experiments going through C^{α}.

Experiment	Dim.<br>B_0/T<br>MAS	Transfer 1	Transfer 2	Transfer 3<br>spectr. width/kHz	direct dimension<br>spectr. width/kHz<br>points/t_{max}	ind. dim. 1<br>spectr. width/kHz<br>points/t_{max}	ind. dim. 2<br>d1/s<br>points/t_{max}	NS<br><br>time/h	peaks
u-^{15}N,GYFR-^{13}C-labeled sample									
NCACX	3D<br>14.1<br>15	CP H-N, 1.1 ms<br>^{1}H: 71 kHz (ramp)<br>^{15}N: 54 kHz	CP N-CA, 7 ms<br>^{15}N: 11 kHz<br>^{13}C: 4 kHz	C-C DARR, 150 ms<br>^{1}H RF: 15 kHz	^{13}C<br>50 kHz<br>1700/17 ms	^{15}N<br>4 kHz<br>56/13.7 ms	^{13}CA<br>6 kHz<br>44/7.1 ms	8<br>1.9 s<br>45 h	310
NCOCX	3D<br>14.1<br>15	CP H-N, 1.1 ms<br>^{1}H: 71 kHz (ramp)<br>^{15}N: 54 kHz	CP N-CA, 7 ms<br>^{15}N: 11 kHz<br>^{13}C: 4 kHz	C-C DARR, 150 ms<br>^{1}H RF: 15 kHz	^{13}C<br>50 kHz<br>1700/17 ms	^{15}N<br>3.7 kHz<br>48/12.7 ms	^{13}CO<br>6 kHz<br>50/8.1 ms	8<br>1.9 s<br>44 h	310
coNCA	2D<br>14.1<br>15	CP H-C, 4 ms<br>^{1}H: 88 kHz (ramp)<br>^{13}C: 71 kHz	CP CO-N, 7 ms<br>^{15}N: 11 kHz<br>^{13}C: 4 kHz	CP N-CA, 7 ms<br>^{15}N: 11 kHz<br>^{13}C: 4 kHz	^{13}C<br>40.3 kHz<br>1370/17 ms	^{15}N<br>2.5 kHz<br>40/15.6 ms	-	512<br>1.8 s<br>21 h	12
caNCO	2D<br>14.1<br>15	CP H-C, 1 ms<br>^{1}H: 88 kHz (ramp)<br>^{13}C: 71 kHz	CP CA-N, 6 ms<br>^{15}N: 11 kHz<br>^{13}C: 4 kHz	CP N-CO, 6.5 ms<br>^{15}N: 11 kHz<br>^{13}C: 4 kHz	^{13}C<br>50 kHz<br>1500/15 ms	^{15}N<br>2.5 kHz<br>40/15.6 ms	-	128<br>1.7 s<br>3.6 h	14
u-^{15}N,ILV-^{13}C–labeled sample									
NCACX	3D<br>14.1<br>15	CP H-N, 1.1 ms<br>^{1}H: 71 kHz (ramp)<br>^{15}N: 54 kHz	CP N-CA, 6.5 ms<br>^{15}N: 11 kHz<br>^{13}C: 4 kHz	C-C DARR, 150 ms<br>^{1}H RF: 15 kHz	^{13}C<br>50 kHz<br>1500/15 ms	^{15}N<br>3.6 kHz<br>30/8 ms	^{13}CA<br>6 kHz<br>36/5.8 ms	24<br>1.98 s<br>62 h	195
NCOCX	3D<br>14.1<br>15	CP H-N, 1.1 ms<br>^{1}H: 71 kHz (ramp)<br>^{15}N: 54 kHz	CP N-CA, 7 ms<br>^{15}N: 11 kHz<br>^{13}C: 4 kHz	C-C DARR, 150 ms<br>^{1}H RF: 15 kHz	^{13}C<br>50 kHz<br>1700/17 ms	^{15}N<br>3 kHz<br>26/8.3 ms	^{13}CO<br>3 kHz<br>20/6.3 ms	8<br>1.7 s<br>33.5 h	237
coNCA	2D<br>14.1<br>15	CP H-C, 4 ms<br>^{1}H: 88 kHz (ramp)<br>^{13}C: 71 kHz	CP CO-N, 7 ms<br>^{15}N: 11 kHz<br>^{13}C: 4 kHz	CP N-CA, 7 ms<br>^{15}N: 11 kHz<br>^{13}C: 4 kHz	^{13}C<br>40.3 kHz<br>1370/17 ms	^{15}N<br>2.5 kHz<br>40/15.6 ms	-	8<br>1.8 s<br>21 h	8
CCCdarrdarr	3D<br>14.1<br>15	CP H-C, 4 ms<br>^{1}H: 88 kHz (ramp)<br>^{13}C: 71 kHz	C-C DARR, 120 ms<br>^{1}H RF: 12 kHz	C-C DARR, 120 ms<br>^{1}H RF: 12 kHz	^{13}C<br>40.3 kHz<br>1210/15 ms	^{13}C<br>30 kHz<br>130/4.3 ms	^{13}C<br>30 kHz<br>136/4.3 ms	2<br>1.15 s<br>49 h	267
u-^{15}N,LKP-^{13}C-labeled sample									
NCACX	3D<br>14.1<br>15	CP H-N, 1.1 ms<br>^{1}H: 71 kHz (ramp)<br>^{15}N: 54 kHz	CP N-CA, 5.5 ms<br>^{15}N: 11 kHz<br>^{13}C: 4 kHz	C-C DARR, 80 ms<br>^{1}H RF: 15 kHz	^{13}C<br>50 kHz<br>1500/15 ms	^{15}N<br>3.6 kHz<br>56/11 ms	^{13}CA<br>6 kHz<br>52/8.5 ms	8<br>1.8 s<br>54 h	145
NCOCX	3D<br>14.1<br>15	CP H-N, 1.1 ms<br>^{1}H: 71 kHz (ramp)<br>^{15}N: 54 kHz	CP N-CA, 5.5 ms<br>^{15}N: 11 kHz<br>^{13}C: 4 kHz	C-C DARR, 80 ms<br>^{1}H RF: 15 kHz	^{13}C<br>50 kHz<br>1500/15 ms	^{15}N<br>4.6 kHz<br>56/11.9 ms	^{13}CO<br>6 kHz<br>46/7.5 ms	8<br>1.8 s<br>43.8 h	237
coNCA	2D<br>14.1<br>15	CP H-C, 3 ms<br>^{1}H: 85 kHz (ramp)<br>^{13}C: 70 kHz	CP CO-N, 7 ms<br>^{15}N: 11 kHz<br>^{13}C: 4 kHz	CP N-CA, 7 ms<br>^{15}N: 11 kHz<br>^{13}C: 4 kHz	^{13}C<br>40.3 kHz<br>1370/17 ms	^{15}N<br>3.2 kHz<br>40/12.2 ms	-	576<br>1.7 s<br>23.5 h	25

Supplementary Table 4: Acquisition parameters of MAS NMR spectra for resonance assignment using proton-detected experiments on u-[^{2}H,^{13}C,^{15}N]-labeled sample, back-exchanged to 100% in H_2O-buffer. The hcaCBcaNH experiment has not been used for the automatic assignment, but only collected afterwards to confirm assignments manually.

Experiment	Dim.<br>B_0/T<br>MAS	Transfer 1	Transfer 2	Transfer 3	Transfer 4	direct dimension<br>spectr. width/kHz<br>points/t_{max}	ind. dim. 1<br>spectr. width/kHz<br>points/t_{max}	ind. dim. 2<br>spectr. width/kHz<br>points/t_{max}	NS<br>d1/s<br>time/h	peaks
hNH	2D	CP H-N, 1 ms	CP N-H, 1 ms	-	-	^{1}H	^{15}N		128	146
	14.1	^{1}H: 95 kHz (ramp)	^{1}H: 95 kHz (ramp)			20.1 kHz	3.04 kHz		0.95 s	
	55	^{15}N: 40 kHz	^{15}N: 40 kHz			1210/30 ms	112/36.5 ms		8 h	
hCONH	3D	CP H-CO, 3 ms	CP CO-N, 6 ms	CP N-H, 1 ms	-	^{1}H	^{15}N	^{13}CO	128	304
	14.1	^{1}H: 95 kHz (ramp)	^{15}N: 22 kHz (ramp)	^{1}H: 95 kHz (ramp)	-	20.1 kHz	2.8 kHz	3 kHz	0.95 s	
	38	^{15}N: 40 kHz	^{13}C: 13 kHz	^{1}N: 40 kHz	-	1210/30 ms	46/16 ms	50/16.3 ms	44 h	
hCANH	3D	CP H-CO, 3 ms	CP CO-N, 6 ms	CP N-H, 1 ms	-	^{1}H	^{15}N	^{13}CO	16	255
	14.1	^{1}H: 95 kHz (ramp)	^{15}N: 22 kHz (ramp)	^{1}H: 95 kHz (ramp)	-	20.1 kHz	2.8 kHz	3 kHz	1.04 s	
	38	^{15}N: 40 kHz	^{13}C: 13 kHz	^{1}N: 40 kHz	-	1210/30 ms	46/16 ms	68/9.6 ms	64 h	
hcoCAcoNH	3D	CP H-CO, 3 ms	CO-CA out/back	CO-N CP	CP N-H, 1 ms	^{1}H	^{15}N	^{13}CA	16	169
	14.1	^{1}H: 95 kHz (ramp)	INEPT	^{15}N: 22 kHz (ramp)	^{1}H: 95 kHz (ramp)	20.1 kHz	2.3 kHz	15 kHz	1.1 s	
	38	^{15}N: 40 kHz	delay 5 ms	^{13}C: 13 kHz	^{1}N: 40 kHz	1210/30 ms	40/17 ms	106/7.0 ms	92 h	
hcaCBcaNH	3D	CP H-CA, 4 ms	CA-CB out/back	CA-N CP	CP N-H, 1 ms	^{1}H	^{15}N	^{13}CA	16	not
	14.1	^{1}H: 85 kHz (ramp)	INEPT	^{15}N: 38 kHz (ramp)	^{1}H: 82.5 kHz (ramp)	20.1 kHz	2.55 kHz	11.3 kHz	1.1 s	used in
	53	^{15}N: 33 kHz	delay 6.5 ms	^{13}C: 14 kHz	^{1}N: 38 kHz	1210/30 ms	64/25 ms	192/8.4 ms	58 h	FLYA

Supplementary Table 5: Acquisition parameters of experiments used for distance restraint measurements.

Experiment	Dim.<br>B_0/T<br>sample	Through-bond<br>transfer	Through-space<br>transfer	direct dimension<br>spectr. width/kHz<br>points/t_{max}	ind. dim. 1<br>spectr. width/kHz<br>points/t_{max}	ind. dim. 2<br>spectr. width/kHz<br>points/t_{max}	NS<br>d1/s<br>time/h
h(N/C)h-RFDR-h(N/C)-H	3D	CP H-N & H-C, 2.5 ms	^{1}H RFDR, 8 ms (440 cycles)	^{1}H	^{15}N & ^{13}C	^{15}N & ^{13}C	4
MAS NMR	14.1	^{1}H: 95 kHz (ramp)	100 kHz ^{1}H pulses	12.02 kHz	6.8 kHz	6.8 kHz	0.83 s
MAS: 55 kHz	sample 6	^{15}N/^{13}C: 41/43 kHz		1200/50 ms	164/24.1 ms	168/24.7 ms	118 h
LKP ^{13}C DARR	2D	CP H-C, 1.6 ms	^{13}C-^{13}C DARR, 350 ms	^{13}C	^{13}C	-	192
MAS NMR	14.1	^{1}H: 70 kHz (ramp)	^{1}H RF: 12 kHz	83.3 kHz	30 kHz		1.6 s
MAS: 13 kHz	sample 4	^{13}C: 55 kHz		2500/15 ms	200/6.6 ms		42 h
H-H-C NOESY	3D	HMQC type	^{1}H-^{1}H NOESY, 400 ms	^{1}H	^{1}H	^{13}C	12
solution-state	18.8	delay 4 ms		8 kHz	1.5 kHz	3.6 kHz	1.6 s
NOESY-HMQC	sample 7			560/70 ms	32/20 ms	72/20 ms	64 h
H-C-C NOESY	3D	HMQC type	^{1}H-^{1}H NOESY, 350 ms	^{1}H	^{13}C	^{13}C	4
solution-state	22.3	delay 4 ms		11.4 kHz	4.8 kHz	4.8 kHz	1.65 s
HMQC-NOESY-HMQC	samples 8-10, see Figure 4E			571/50 ms	95/20 ms	70/14.7 ms	60 h

Supplementary Table 6: Refinement of TET2 structures with *phenix.real_space_refine*. Shown are structure statistics obtained when using the three different EM maps (at 4.1, 6 or 8 Å resolution, respectively), and using as starting models different structures, either the crystal structure, or the structures obtained from the NMR and EM approach, that used 4.1, 6 or 8 Å resolution EM maps, respectively.

Model		RMSD w.r.t. PDB 1y0r all atoms	Cα only	CC_{mask}	Ramachandran favored region (%)	All-atom molprobity clashscore	Rotamer outliers (%)
Biological unit derived from X-ray structure (1y0r)		-	-	0.7836	97.24	6.08	2.21
Refinement of biological unit derived from X-ray structure (1y0r) in cryo-EM map at 4.1 Å , using phenix.real_space_refine (simulated annealing and minimization)		0.739	0.583	0.8597	92.71	6.77	11.03
After Steps 1, 2, 3 with 4.1 Å EM map. PDB 6F3K		1.06	0.736	0.6668	92.02	161.29	8.56
After Steps 1, 2, 3 at 4.1 Å , refined in cryo-EM map at 4.1 Å , using phenix. real_space_refine	Local-grid-search and minimization.	1.087	0.74	0.6846	89.74	1.71	1.03
	Morphing and minimization.	1.058	0.737	0.7036	90.03	12.07	8.22
	Simulated annealing and minimization. PDB 6R8N	1.025	0.748	0.728	90.03	3.16	26.03
After Steps 1, 2, 3 with 6 Å EM map		2.467	2.139	0.418	81.48	33.93	38.24
After Steps 1, 2, 3 with 6 Å EM map refined in cryo-EM map at 6 Å using phenix.real_space_refine (sim. annealing and minimization) (sim. annealing and minimization)		2.157	1.736	0.5478	82.62	5.01	0.34
After Steps 1, 2, 3 with 6 Å EM map refined in cryo-EM map at 4.1 Å using phenix.real_space_refine (sim. annealing and minimization)		2.112	1.811	0.5553	83.19	5.61	0.34
(After Steps 1, 2, 3 with 8 Å EM map		3.082	2.544	0.227	83.52	77.46	44.52
(After Steps 1, 2, 3 with 8 Å EM map refined in cryo-EM map at 8 Å using phenix.real_space_refine (sim. annealing and minimization)		3.111	2.612	0.4611	82.62	6.35	0.34
(After Steps 1, 2, 3 with 8 Å EM map refined in cryo-EM map at 4.1 Å using phenix.real_space_refine (sim. annealing and minimization)		3.262	2.834	0.4803	82.34	5.9	0

References

1. Shen, Y. & Bax, A. Protein backbone and sidechain torsion angles predicted from NMR chemical shifts using artificial neural networks. *J. Biomol. NMR* **56**, 227–241 (2013).

2. Schmidt, E. & Guntert, P. A new algorithm for reliable and general NMR resonance assignment. *J. Am. Chem. Soc.* **134**, 12817–12829 (2012).

3. Matthew L. Baker, C. F. H. T. J., Mariah R. Baker & Chiu, W. Gorgon and pathwalking: Macromolecular modeling tools for subnanometer resolution density maps. *Biopolymers* **97**, 655–668 (2012).

4. Kucukelbir, A., Sigworth, F. J. & Tagare, H. D. Quantifying the local resolution of cryo-em density maps. *Nat. Meth.* **11**, 63 (2014).

5. Gans, P. *et al.* Stereospecific isotopic labeling of methyl groups for NMR spectroscopic studies of high-molecular-weight proteins. *Angew. Chem. Int. Ed.* **49**, 1896 (2010).
