# Supplementary material for: Integrated NMR and cryo-EM atomic-resolution structure determination of a half-megadalton enzyme complex
Source: Nat Commun. 2019 Jun 19;10:2697. doi: 10.1038/s41467-019-10490-9 (PMC6584647; doi:10.1038/s41467-019-10490-9)
Supplement: Supplementary file 2 — Peer Review File [file 41467_2019_10490_MOESM2_ESM.pdf]

Editorial Note: This manuscript has been previously reviewed at another journal that is not operating a transparent peer review scheme. The manuscript was considered suitable for publication without further review at *Nature Communications.*
